# Aurora-A-mediated cytosolic localization of Maf1 promotes cell proliferation via regulating mitochondrial function in HCC

**DOI:** 10.1038/s41420-025-02885-z

**Published:** 2025-12-03

**Authors:** Shiang-Jie Yang, Yu-Heng Kuan, Zong-Xin Ooi, Hong-Sheng Lai, Hsin-Yi Wu, Pao-Chi Liao, Yih-Jyh Lin, Che Min Su, Ming-Derg Lai, Liang-Yi Hung

**Affiliations:** 1 The Institute of Basic Medical Sciences, College of Medicine, National Cheng Kung University, Tainan, 70101 Taiwan; 2 Department of Biotechnology and Bioindustry Sciences, College of Bioscience and Biotechnology, National Cheng Kung University, Tainan, 70101 Taiwan; 3 Instrumentation Center, National Taiwan University, Taipei, 106 Taiwan; 4 Department of Environmental and Occupational Health, College of Medicine, National Cheng Kung University, Tainan, 704 Taiwan; 5 Department of Surgery, National Cheng Kung University Hospital, College of Medicine, National Cheng Kung University, Tainan, 70101 Taiwan; 6 Department of Pharmacology, College of Medicine; National Cheng Kung University, Tainan, 70101 Taiwan; 7 University Center for Bioscience and Biotechnology, National Cheng Kung University, Tainan, 70101 Taiwan; 8 Graduate Institute of Medicine, College of Medicine, Kaohsiung Medical University, Kaohsiung, 80708 Taiwan; 9 Present Address: Department of Cell Biology, Albert Einstein College of Medicine, Bronx, NY 10461 USA

**Keywords:** Cancer, Cell biology

## Abstract

Maf1 is a well-known RNA polymerase III repressor and functions as a tumor suppressor due to its role in inhibiting tRNA synthesis. However, the role of Maf1 in hepatocellular carcinoma (HCC) remains unclear. This study identified Aurora-A as a novel upstream regulator of Maf1 in HCC. We demonstrated that Aurora-A interacts with the C domain of Maf1 and phosphorylates it at Threonine-212, leading to increased protein stability and cytosolic accumulation of Maf1. Importantly, the Aurora-A-enhanced cytosolic localization of Maf1 promotes mitochondrial dysfunction and glycolytic activity, ultimately driving HCC cell proliferation. In contrast, mutation of the Thr-212 site abolishes these effects, confirming its critical role. Significantly, elevated Maf-1 expression correlates with unfavorable clinical outcomes in HCC, particularly among patients with high Aurora-A expression. Furthermore, HCC cells with overexpressed Maf1 have heightened sensitivity to Aurora-A inhibitors, suggesting a potential therapeutic vulnerability. Our study uncovers a non-canonical, oncogenic role of Maf1 in HCC and highlights the Aurora-A-Maf1 axis as a promising target for personalized cancer therapy.

## Introduction

The efficiency of transfer RNA (tRNA) biosynthesis is crucial for various cellular processes, including protein synthesis, cell cycle progression, mitochondrial function, and cell proliferation [1, 2]. Dysregulated tRNA biosynthesis has been identified as a significant factor contributing to the onset and pathogenesis of various diseases [3]. The accumulation of RNA polymerase III (Pol III) products has been recognized as a hallmark of various types of cancer [4]. Furthermore, it was reported that modifications and derivatives of tRNA play a critical role in tumorigenesis [5–7]. Alterations in tRNA modifications have been associated with drug resistance, mitochondrial dysfunction, and tumor metastasis in different cancer types, including hepatocellular carcinoma (HCC) [8, 9]. Additionally, tRNA derivatives can promote cell proliferation and cell cycle progression by regulating the expression of Aurora-A during the G0/G1 stage [10, 11]. Despite accumulating evidence supporting the important role of tRNA regulatory mechanisms in tumorigenesis, further studies are required to evaluate whether targeting these pathways is a promising strategy in cancer therapy.

Maf1 is a well-characterized suppressor of RNA polymerase III (Pol III) that regulates tRNA biosynthesis by inhibiting the interaction between Pol III and transcription factors TFIIIB and TFIIIC [12]. Under cellular stress conditions—such as nutrient deprivation, DNA damage, and drug-induced cytotoxicity—Maf1 is dephosphorylated and translocated to the nucleus to repress tRNA synthesis [13, 14]. In contrast, Maf1 remains predominantly phosphorylated and sequestered in the cytoplasm during normal growth, thereby preventing RNA Pol III inhibition [15]. Maf1 polypeptide consists of three domains: domain A, domain B, and domain C [13]. Domains A and B interact with the large subunit of RNA Pol III [16]; domain C regulates the subcellular localization and protein stability of Maf1 [17]. Notably, the mammalian target of rapamycin (mTOR), a conserved serine/threonine kinase, plays a pivotal role in modulating Maf1 function by phosphorylating several sites, including Ser-60, Ser-68, and Ser-75, which in turn influence its localization and activity [18, 19]. In addition, post-translational modifications, such as ubiquitylation and SUMOylation, have been implicated in regulating Maf1 function [20, 21]. Despite the increasing understanding of the regulatory mechanisms that govern Maf1 function, the precise role of Maf1 in tumorigenesis remains to be further elucidated.

Due to its ability to suppress RNA Pol III activity, Maf1 has been proposed as a potential tumor suppressor [22]. For instance, in HCC and prostate cancer, a significant decrease in nuclear Maf1 expression has been observed. The nuclear localization of Maf1 exerts its tumor-suppressive function through various regulatory mechanisms, including the inhibition of tRNA biosynthesis, modulation of lipid metabolism, and promotion of PTEN expression [23, 24]. In contrast, in colorectal cancer (CRC), Maf1 expression is associated with an unfavorable outcome in tumors with microsatellite instability (MSI) [25], highlighting the context-dependent nature of its role in cancer. These findings indicate the complex and multifaceted role of Maf1 in tumorigenesis.

In this study, we investigated the association of Maf1 overexpression and poor prognosis in HCC, a finding that contrasts with previous reports. This discrepancy led us to hypothesize the involvement of novel regulatory factors in modulating Maf1 function. Previous studies have demonstrated that CDK1, a key cell cycle regulator, enhances RNA Pol III activity during cell cycle progression [26, 27], suggesting that cell cycle-associated kinases may contribute to regulating RNA Pol III-mediated transcription. Aurora-A, a serine/threonine kinase, is hyperactivated during the G2/M phase of the cell cycle, where it promotes cell cycle progression [28, 29]. Additionally, accumulating evidence indicates that Aurora-A overexpression plays a critical role in tumor malignancy in various types of cancer [30, 31]. Given those findings, we will further investigate the potential interaction between Aurora-A and MAf1 in regulating HCC progression. Our studies revealed a novel regulatory mechanism involving Aurora-A and its interaction with Maf1. We found that Aurora-A-mediated cytosolic localization of Maf1 promotes cell proliferation and is associated with poor prognoses in HCC patients. In Maf1-overexpressing HCC cells, the treatment of Aurora-A inhibitor MLN8237 showed higher effectiveness in eradicating cancer cells. Our study demonstrates that Aurora-A is a novel kinase in regulating Maf1 function and targeting Aurora-A may be a promising strategy in cancer therapy, particularly for HCC patients with higher expression levels of Maf1 and Aurora-A.

## Results

### Elevated expression of Maf1 correlates with poor prognosis in HCC patients

**Fig. 1:**
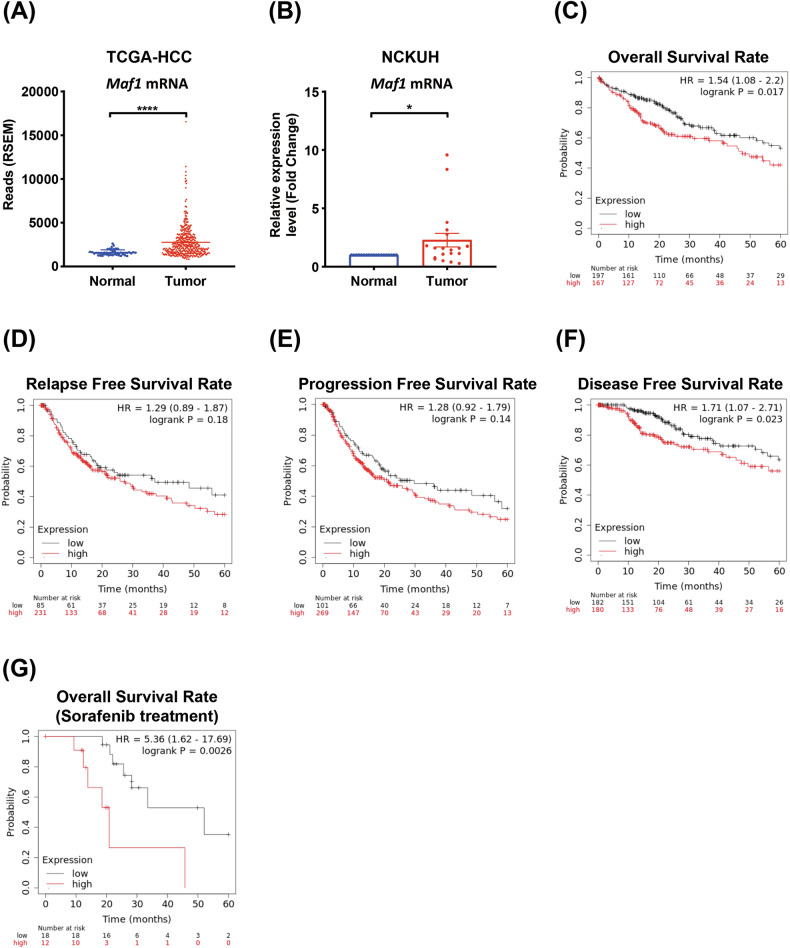
Elevated expression of *Maf1* mRNA correlates with poor clinical outcomes in HCC patients. **A** The expression of *Maf1* mRNA in tumor tissues (red) and adjacent normal tissues (blue) of HCC patients was analyzed using the TCGA-HCC dataset. FPKM analysis was conducted, and statistical significance was determined using Student’s *t*test. ****, *p* < 0.0001. **B** *Maf1* mRNA expression in tumor tissues (red) and adjacent normal tissues (blue) of HCC patients from NCKUH was analyzed by RT-qPCR, and statistical significance was determined using Student’s *t* test. *, *p* < 0.05. **C-F** Clinical outcomes of HCC patients stratified based on high (red) or low (black) *Maf1* mRNA expression were analyzed using the Kaplan-Meier plotter (https://kmplot.com/analysis/). Survival curves of overall survival (**C**), relapse-free survival (**D**), progression-free survival (**E**), and disease-free survival rates (**F**) were shown. **G** Kaplan-Meier plot showing overall survival rate in HCC patients who were received sorafenib treatment.

Given the conflicting reports on the dual roles of Maf1 in tumorigenesis, we analyzed the expression of *Maf1* mRNA in various cancers using the TCGA datasets. Compared to adjacent tissues, we found that *Maf1* mRNA was upregulated in cholangiocarcinoma (CHOL), liver hepatocellular carcinoma (LIHC), and glioblastoma multiforme (GBM) (Fig. 1A and Supplementary Fig. S1). To further confirm this result, the tumors and their adjacent normal tissues of HCC patients from the National Cheng Kung University Hospital (NCKUH) were collected to analyze the expression of *Maf1* mRNA by RT-qPCR. The results showed that *Maf1* mRNA is up-regulated in the HCC tumors compared to their adjacent normal tissues (Fig. 1B). Significantly, the elevated *Maf1* mRNA in HCC patients is associated with poor prognoses (Fig. 1C–F).

Although new therapeutic strategies have emerged, sorafenib remains a treatment option for advanced HCC [32–34]. Next, we evaluated the effect of elevated Maf1 in response to sorafenib treatment. The result indicated that HCC patients who received sorafenib treatment with higher Maf1 expression exhibited a poor prognosis (Fig. 1G). These results suggest that Maf1 may exert an unknown function in promoting HCC progression that is inconsistent with the previous reports [22].

### Aurora-A interacts with the C domain of Maf1 and regulates its subcellular localization and function

**Fig. 2:**
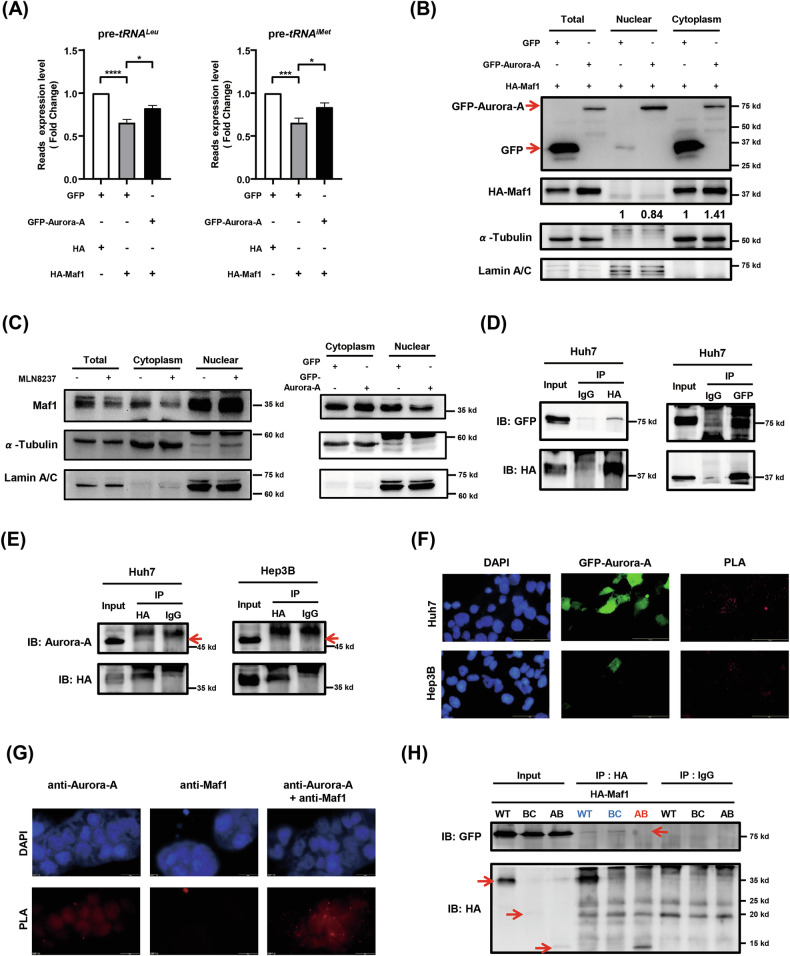
Aurora-A serves as a novel regulator of Maf1 function and interacts with the C-domain of Maf1. **A-B** Hep3B (A) and Huh7 (B) cells were transiently transfected with vector control (GFP or HA), GFP-Aurora-A, and HA-Maf1. **A** RT-qPCR analyzed the expression of pre-*tRNA*^*Leu*^ (left) and pre-*tRNAi*^*Met*^ (right). Statistical analysis was performed using Student’s *t*test. *, *p* < 0.05; ***, *p* < 0.001; ****, *p* < 0.0001. **B** Nuclear and cytoplasmic fractions were purified to determine the subcellular localization of HA-Maf1 by western blot analysis. **C** Left: Hep3B cells were treated with (+) or without (–) 500 nM MLN8237 for 6 h. Nuclear and cytoplasmic fractions were purified to determine the subcellular localization of endogenous Maf1 by western blot analysis. Right: Hep3B cells were transfected with GFP-Aurora-A, and nuclear and cytoplasmic fractions were purified to determine the subcellular localization of endogenous Maf1 by western blot analysis. α-Tubulin is the cytosol fraction marker, and Lamin A/C is the nucleus fraction marker. **D** Huh7 cells were transiently co-transfected with GFP-Aurora-A and HA-Maf1. The interaction between GFP-Aurora-A and HA-Maf1 was determined by co-immunoprecipitation (co-IP) assay using anti-HA (left) or anti-GFP (right) antibodies. **E** Huh7 (left) and Hep3B (right) cells were transiently transfected with HA-Maf1. Co-IP assay was performed to identify the interaction between endogenous Aurora-A and HA-Maf1 using anti-HA antibody. IgG was used as an IP negative control. Red arrows indicate endogenous Aurora-A. **F** Huh7 and Hep3B cells were transiently transfected with GFP-Aurora-A and HA-Maf1. Interaction between HA-Maf1 and GFP-Aurora-A was determined by in situ Proximity Ligation Assay (PLA) using anti-HA and anti-GFP antibodies. Red dots (PLA) indicate the interaction between HA-Maf1 and GFP-Aurora-A. **G** The interaction between endogenous Aurora-A and Maf1 was determined by in situ PLA using specific anti-Aurora-A and anti-Maf1 antibodies in Hep3B cells. Red dots (PLA) indicate the interaction between endogenous Aurora-A and Maf1. DAPI is a DNA-specific dye. **H** Huh7 cells were transiently transfected with GFP-Aurora-A and HA-Maf1/WT, or HA-Maf1/BC, or HA-Maf1/AB. Co-IP analysis was performed to identify the interaction between GFP-Aurora-A and HA-Maf1 using anti-HA antibodies. IgG was used as an IP negative control.

It was reported that Aurora-A is a biomarker for predicting the effect of sorafenib treatment [35], and Aurora-A inhibitor can enhance the drug sensitivity of sorafenib in HCC [36]. Here, we tested the possibility of Aurora-A in regulating the oncogenic function of Maf1 in HCC. We first examined the effect of Aurora-A in regulating the activity of RNA polymerase III (Pol III). The results indicated that overexpression of Aurora-A can restore Maf1-mediated suppression of tRNA expression in HCC cells (Fig. 2A), suggesting that Aurora-A can negatively regulate Maf1-mediated RNA Pol III activity suppression. Previous studies have shown that phosphorylation and its cytosolic sequestration can abolish the Maf1-mediated suppression of RNA Pol III activity [37]. Our results showed that overexpression of Aurora-A promotes the cytosolic localization of Maf1 in HCC cells (Fig. 2B). To further analyze the effect of Aurora-A in regulating the subcellular localization of endogenous Maf1, a cell fractionation experiment was performed, followed by western blot analysis. The results showed that cell treated with Aurora-A inhibitor MLN 8237 decreases the Aurora-A kinase activity (Supplementary Figure S2) and the cytosolic localization of endogenous Maf1 (Fig. 2C, left); overexpression of GFP-Aurora-A resulted in an obvious decrease in nuclear localization of endogenous Maf1 (Fig. 2C, right). Both the co-immunoprecipitation assay and the in situ PLA demonstrated that Aurora-A directly interacts with Maf1 in HCC cells (Fig. 2D–G). Previous studies indicated that the Maf1 protein contains three conserved domains: A, B, and C [12, 13] (Supplementary Fig. S3A). To map the interaction domain between Aurora-A and Maf1, three constructs containing different domains of Maf1, namely HA-Maf1-A, HA-Maf1-BC, and HA-Maf1-AB, were generated (Supplementary Fig. S3A). Western blot confirmed the expression of these constructs, and we found that HA-Maf1-A was not detectable (Supplementary Fig. S3B). Co-immunoprecipitation assay indicated that Aurora-A could only interact with HA-Maf1-WT and HA-Maf1-BC, but not HA-Maf1-AB (Fig. 2H). This result indicated that the C-domain of Maf1 is essential for its interaction with Aurora-A. Interestingly, this interaction between Maf1 and Aurora-A is slightly attenuated in the kinase-dead mutant of Aurora-A (Supplementary Fig. S4). These results indicate that Aurora-A can interact with Maf1 by the C-domain and regulate its ability to modulate tRNA biosynthesis.

### Aurora-A enhances Maf1 protein stability

**Fig. 3:**
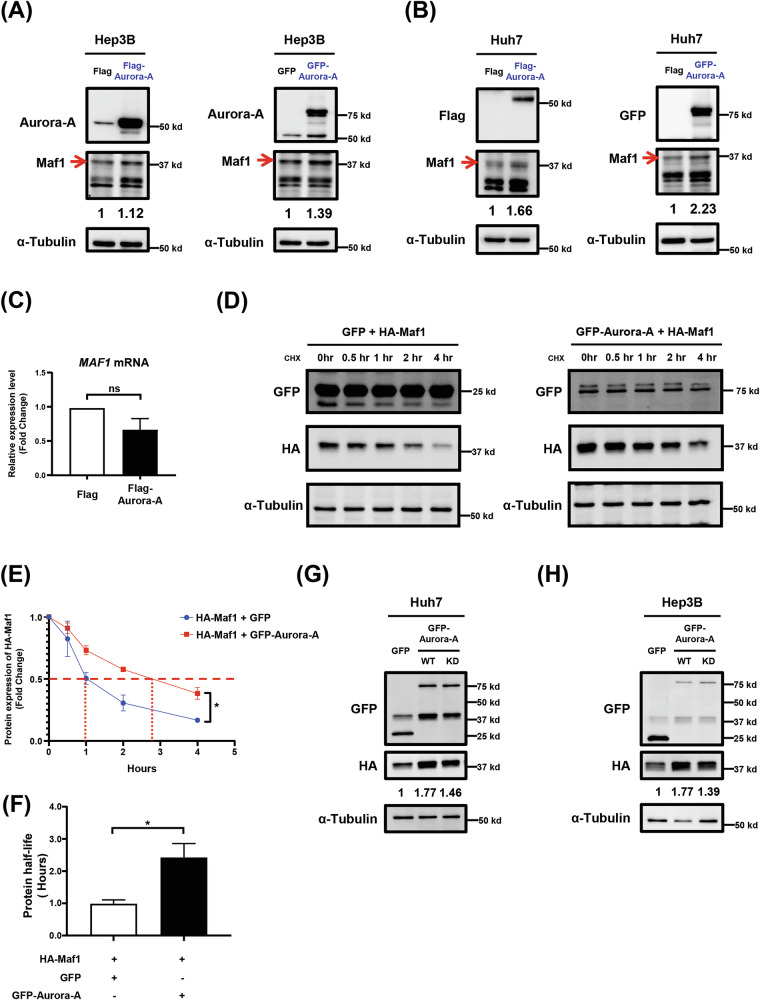
Aurora-A enhances Maf1 protein stability in a kinase activity-dependent manner. **A**-**B** Hep3B (**A**) and Huh7 (**B**) cells were transiently transfected with Flag-Aurora-A or GFP-Aurora-A. The protein expression of Maf1 was analyzed by western blot. **C** Expression of Maf1 mRNA was analyzed in Huh7 cells following transient transfected with Flag vector control or Flag-Aurora-A. **D**-**F** Huh7 cells were transiently co-transfected with HA-Maf1 and GFP vector control (left) or GFP-Aurora-A (right). At 24 hours of post transfection,cells were treated with cycloheximide for various time periods. The expression of GFP, GFP-Aurora-A, and HA-Maf1 was analyzed by western blot (**D**). The relative expression levels of HA-Maf1 protein at each time points were quantified and compared to the 0-hour time point (**E**). The protein half-life of HA-Maf1 was shown (**F**). Statistical analysis was performed using Two-way ANOVA. *, p 0.05. **G**-**H** Huh7 (**G**) and Hep3B (**H**) cells were transiently transfected with HA-Maf1 and GFP vector control or GFP-Aurora-A/WT or GFP-Aurora-A/KD (kinase dead). The protein expression of HA-Maf1 was subsequently analyzed by western blot. α-Tubulinis a loading control for western blot.

In addition to the direct interaction between Aurora-A and Maf1, our results showed that overexpression of GFP-Aurora-A leads to an increased protein level of Maf1 (Fig. 2B, lane 2). This phenomenon is further confirmed in Flag-Aurora-A-overexpressing Hep3B and Huh7 cells (Fig. 3A, B), and overexpression of Aurora-A did not alter the mRNA stability of *Maf1* mRNA (Fig. 3C). We found that overexpressed Aurora-A can prolong the protein stability of Maf1 (Fig. 3D–F); whereas, knocking down the expression of Aurora-A by shAurora-A decreased the expression level of Maf1, and the addition of MG132 restored the protein level of Maf1 (Supplementary Fig. S5). Interestingly, inhibition of Aurora-A kinase activity using Aurora-A inhibitor MLN8237 (Fig. 2C) or overexpression of Aurora-A kinase-dead mutant (KD) (Fig. 3G, H) attenuated its ability to enhance the protein level of Maf1. These results suggested that Aurora-A can enhance Maf1 protein stability in HCC cells in a kinase activity-dependent manner.

### The phosphorylation of Thr-212 on Maf1 is essential for maintaining its protein stability and cytosolic localization in HCC cells

**Fig. 4:**
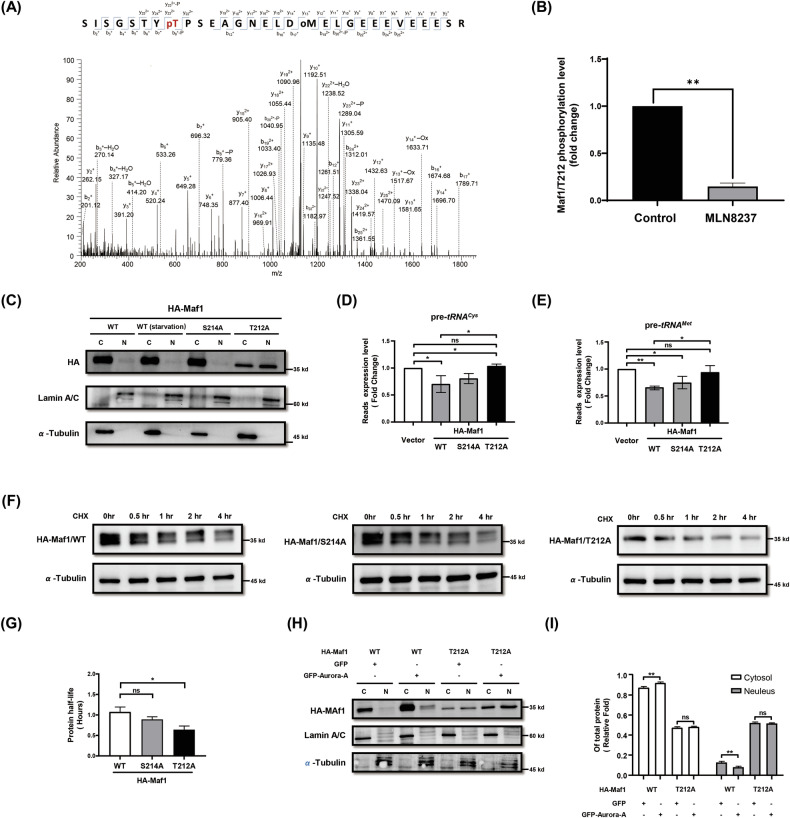
Phosphorylation of Maf1 Thr-212 is crucial for maintaining its protein stability and cytosolic localization. **A** MS/MS spectrum of the MAF1 peptide (residues 205–234, SISGSTYpTPSEAGNELDMELGEEEVEEESR) generated by HCD fragmentation. The observed y222^+^ (1247.51 Da) and y232^+^ (1338.04 Da) ions indicate that T212 is phosphorylated. Phosphorylation and oxidation were denoted as “p” and “o” on the corresponding residues. **B** Hep3B cells transfected with HA-Maf1 were treated with or without (control) 500 nM MLN8237 for 6 h. HA-Maf1 was IP using anti-HA antibodies and subjected to LC–MS/MS analysis. The phosphorylation levels of Maf1 T212 were quantified and presented as fold change. **C** Huh7 cells were transiently transfected with HA-Maf1/WT, /HA-Maf1/T212A, or /HA-Maf1/S214A. Western blot analysis was performed to determine the nuclear (N) and cytoplasmic (**C**) localization of Maf1. α-Tubulin was used as a cytoplasmic marker, and Lamin A/C was a nuclear marker. **D-E** Huh7 cells were transiently transfected with HA, HA-Maf1/WT, HA-Maf1/T212A, or HA-Maf1/S214A. The expression levels of pre-*tRNA*^*Cys*^ (**D**) and pre-*tRNA*^*Met*^ (**E**) were analyzed by RT-qPCR. Statistical analysis was performed using Student’s *t* test. *, *p* < 0.05; **, *p* < 0.001. **F-G** Huh7 cells were transiently transfected with HA-Maf1/WT, HA-Maf1/T212A, or HA-Maf1/S214A. The cells were treated with cycloheximide for various periods (0–4 h), and the protein expression of HA-Maf/WT (upper), HA-Maf/T212A (middle), and HA-Maf/S214A (lower) was analyzed by western blot. The quantification of HA-Maf protein levels (from F) was analyzed to show the protein half-life (**G**). **H–I** Huh7 cells were transiently co-transfected with GFP-Aurora-A and HA-Maf1/WT or HA-Maf1/T212A. The nuclear (N) and cytoplasmic (**C**) localization of Maf1 was evaluated by western blot (H). α-Tubulin is the cytosol fraction marker, and Lamin A/C is the nucleus fraction marker. The quantification results of HA-Maf1 in the cytosol and nucleus were presented as a percentage of the total protein levels (**I**). Statistical analysis was performed using Student’s *t*test. **, *p* < 0.01.

Considering the phosphorylation status of Maf1 can modulate its subcellular localization and the RNA Pol III suppressive activity, we proposed that Aurora-A may exert a regulatory function on Maf1 through phosphorylation. Previous studies have demonstrated that the C-domain of Maf1 is crucial for its cytosolic localization, protein stability, and RNA Pol III suppression [17]. Additionally, our previous result indicated that Aurora-A interacts with the C-domain of Maf1 in HCC cells (Fig. 2H). Therefore, we hypothesized that Aurora-A may regulate Maf1 by phosphorylating the nearby region of the C-domain. Based on the previous studies and the PhosphoSitePlus database [37–40], Thr-212 and Ser-214 of Maf1 were identified as the Aurora-A potential phosphorylation sites. To determine the phosphorylation site of Maf1, we performed liquid chromatography-tandem mass spectrometry (LC-MS/MS) analysis. A phosphorylated Maf1 peptide spanning residues 205–234 (SISGSTYpTPSEAGNELDMELGEEEVEEESR) was detected at [M + 3H]^3+^, m/z 1124.1278 (0.38 ppm), and phosphorylation at T212 (pT212) was confirmed by the presence of the y22²⁺ (1247.51 Da) and y23²⁺ (1338.04 Da) fragment ions (Fig. 4A). Importantly, the relative phosphorylation level of T212 (pT212) was decreased upon Aurora-A inhibitor MLN8237 treatment (Fig. 4B). The abundance of phosphorylated T212 (pT212) was enhanced in Flag-Aurora-A/WT overexpressing cells compared to Flag vector control and Flag-Aurora-A/KD overexpressing cells (Supplementary Fig. S6). Those results indicated that T212 of Maf1 is the phosphorylation site of Aurora-A.

To examine the effect of pT212 on the protein stability, subcellular localization, and the RNA Pol III suppressive function of Maf1, two mutants, Maf1/T212A and Maf1/S214A, were generated. The results showed that Maf1/T212A mutation impedes phosphorylation (Supplementary Fig. S7A) and exhibits a predominant nuclear localization (Fig. 4C). Significantly, the Maf1/T212A mutant lost its function in suppressing tRNA biosynthesis (Fig. 4D, E). Reduced protein stability of the Maf1/T212A mutant, but not the Maf1/WT and Maf1/S214A, was observed (Fig. 4F, G), and the mRNA stability remained unaffected in Maf1/T212A (Supplementary Fig. S7B). Notably, there is a slight disturbance in the interaction between Maf1/T212A and Aurora-A (Supplementary Fig. S8). Subsequently, the essential role of Thr-212 on Maf1 in mediating the regulation of Maf1 cytosolic localization and protein stability by Aurora-A was further elucidated. The results showed that Aurora-A could prolong the protein stability of the Maf1/T212A mutant (Supplementary Fig. S9A–S9C), although the effect was less pronounced compared to wild-type Maf1 (Fig. 3E). Additionally, overexpression of Aurora-A failed to promote the cytosolic localization of the Maf1/T212A mutant (Fig. 4H, I). These results suggested that the phosphorylation at the Thr-212 residue of Maf1 plays an essential role in Aurora-A-mediated regulation of its subcellular localization, protein stability, and function in suppressing RNA pol III activity.

### Cytosolic Maf1 potentially regulates mitochondrial function in HCC

**Fig. 5:**
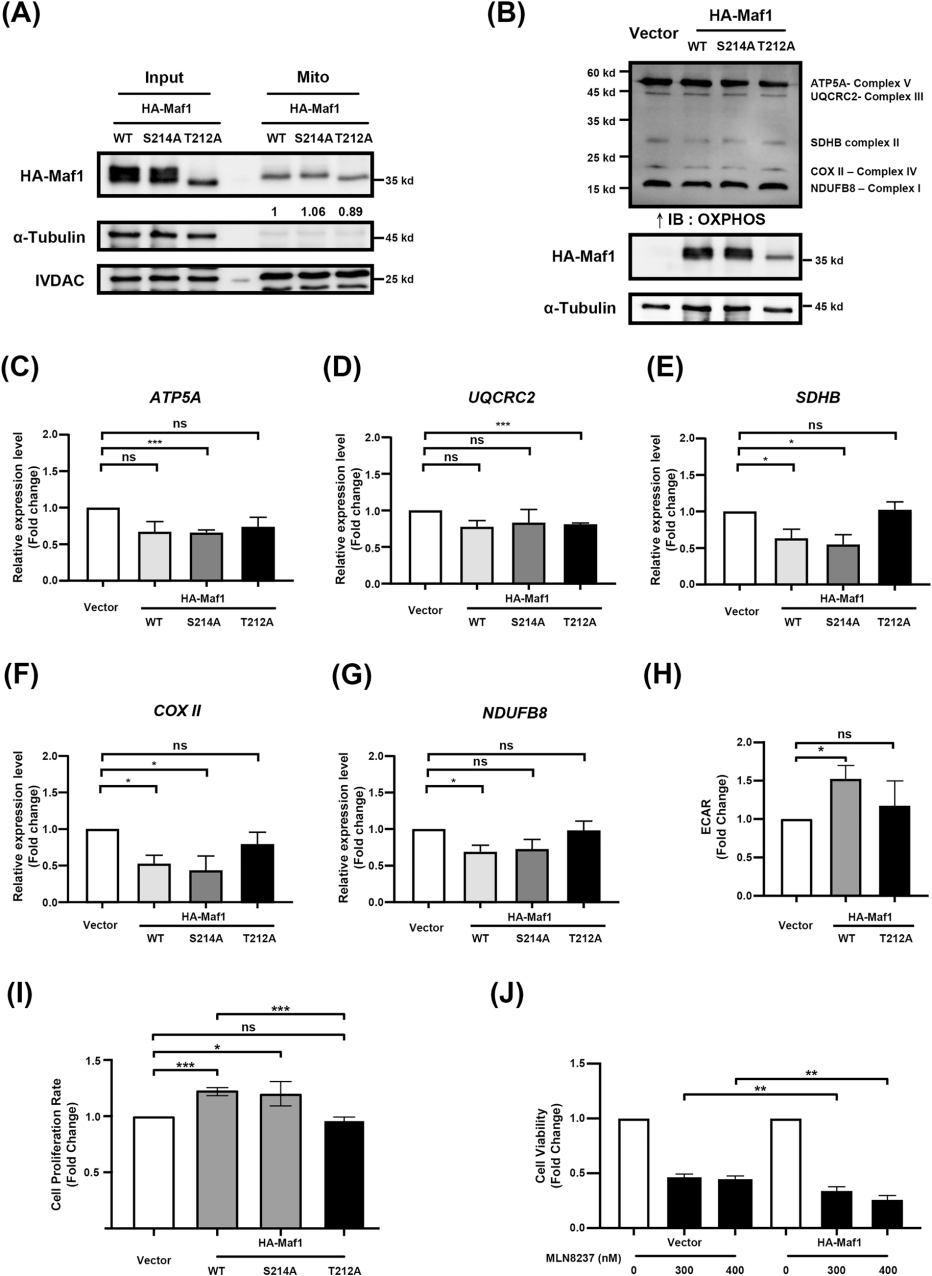
Cytosolic Maf1 promotes cell proliferation by modulating mitochondrial function. **A** Huh7 cells were transiently transfected with HA-Maf1/WT, HA-Maf1/T212A, or HA-Maf1/S214A. Mitochondrial localization of HA-Maf1 was analyzed by western blot. VDAC is a mitochondrial protein, and α-Tubulin is a cytosolic protein. **B–G** Huh7 cells were transiently transfected with HA (vector), HA-Maf1/WT, HA-Maf1/T212A, and HA-Maf1/S214A. Total cell lysates were collected to examine the expression of the five OXPHOS protein complexes by western blot (**B**). The quantitative results were presented as the relative fold change compared to the vector control (**C–G**). Statistical analysis was performed using Student’s *t*test. *, *p* < 0.05; **, *p* < 0.01; ***, *p* < 0.001. **H** Huh7 cells were transiently transfected with HA (vector), HA-Maf1/WT, and HA-Maf1/T212A. The extracellular acidification rate (ECAR) was measured by detecting fluorescence intensity. Thresholds for determining the scope were set based on the increase in fluorescence intensity. Results are presented as the relative fold change compared to the vector control. Statistical analysis was performed using Student’s *t* test. *, *p* < 0.05. **I** Huh7 cells transiently transfected with HA (vector), HA-Maf1/WT, HA-Maf1/T212A, and HA-Maf1/S214A were collected to perform the cell proliferation assay using the CCK-8 assay. The results were presented as the relative fold change compared to the vector control. Statistical analysis was performed using Student’s *t* test. *, *p* < 0.05; ***, *p* < 0.001; ****, *p* < 0.0001; ns, no significance. **J** Huh7 cells transiently transfected with HA (vector) and HA-Maf1/WT were treated with 300 nM or 400 nM MLN8237 for 48 h. Cell viability was assessed using the CCK-8, and the results were presented as the relative fold change compared to the DMSO control. Statistical analysis was performed using Student’s *t-*test. **, *p* < 0.01.

Our results indicated that Aurora-A can regulate the cytosolic accumulation of Maf1 (Figs. 2B, C, and 4H); we hypothesize that the cytosolic localization of Maf1 may have distinct effects on tumorigenesis compared to its nuclear counterpart. Immunofluorescence analysis revealed mitochondrial localization of Maf1 (Supplementary Fig. S10), and cell fractionation assays confirmed that wild-type Maf1 (Maf1/WT), Maf1/T214A, and Maf1/T212A are all present in the mitochondrial fraction (Fig. 5A). These findings suggest that phosphorylation of Maf1 at Thr-212 does not modulate its mitochondrial localization. Mitochondrial dysfunction has been implicated in tumor development [41], and emerging evidence suggests that regulation and modification of mitochondrial tRNA play an important role in maintaining mitochondrial homeostasis [9]. Furthermore, activation of the mitochondrial oxidative phosphorylation system (OXPHOS) has been recognized as an important factor in tumorigenesis [42, 43]. Our studies demonstrated that Maf1 overexpression reduces the levels of OXPHOS complex II (SDHB) (Fig. 5B, E), complex IV (COX II) (Fig. 5B, F), and complex I (NDUFB8) (Fig. 5B, G); without change the levels of complex V (ATP5A) (Fig. 5B, C) and complex III (UQCRC2) (Fig. 5B, D). In contrast, the Maf1/T212A mutant did not affect these complexes (Fig. 5B–G). Previous studies have demonstrated that the downregulation of SDHB can enhance aerobic glycolysis in various cancer types [44, 45]. To investigate the functional role of mitochondrial Maf1, Extracellular Acidification Rate (ECAR) was determined in HCC cells with Maf1/WT and Maf1/T212A expression. The results showed that overexpression of Maf1/WT, but not the Maf1/T212A mutant, significantly increased ECAR (Fig. 5H). Overexpression of Maf1/WT, but not the Maf1/T212A mutant, can significantly promote cell proliferation (Fig. 5H). The impact of Aurora-A-mediated cytosolic localization of Maf1 in HCC was further investigated. The results showed that Maf1-overexpressing HCC cells exhibited increased sensitivity to Aurora-A inhibitors (Fig. 5J). Additionally, HCC patients with high Aurora-A expression and elevated Maf1 in tumor tissues were positively correlated with a poor prognosis (Supplementary Fig. S11). These findings suggested that the Aurora-A-regulated cytosolic localization of Maf1 contributes to tumor progression; in HCC patients with high expression of both Aurora-A and Maf1, targeting Aurora-A could be a promising therapeutic strategy for cancer treatment.

## Discussion

**Fig. 6:**
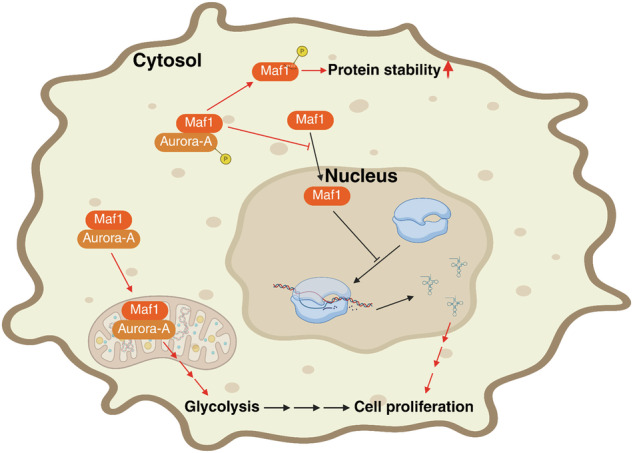
Schematic model illustrating the role of Aurora-A in regulating Maf1 function in HCC cells. In HCC tumors, elevated expression of Aurora-A leads to prolonged protein stability and cytosolic localization of Maf1 through phosphorylation of its Thr-212 residue. The Aurora-A-mediated cytosolic localization of Maf1 promotes cell proliferation by affecting mitochondrial function. The figure was generated using BioRender (https://www.biorender.com/).

The dysregulation of RNA pol III is a well-recognized hallmark in various types of cancer, and targeting the regulatory mechanisms of RNA pol III is considered a promising approach for cancer therapy [46]. Maf1 is recognized as an RNA pol III suppressor and a promising candidate for tumor suppression. Previous reports indicated the multifaceted role of Maf1 in tumorigenesis [23–25], suggesting its complex involvement in tumor development. This study found that *Maf1* mRNA expression is upregulated in HCC and is associated with an unfavorable clinical outcome, and Aurora-A is involved in the modulation of Maf1 phosphorylation in HCC cells. The phosphorylation of Maf1 by Aurora-A regulates its cytosolic localization and protein stability (Fig. 6). The function of Maf1 varies depending on its cytosolic versus nuclear localization. Furthermore, high expression of Maf1 is associated with a poor prognosis in HCC patients with high Aurora-A expression, not in those with low Aurora-A expression. Our findings suggest that the functional role of Maf1 may depend on its subcellular localization in HCC development.

Previous studies demonstrated the role of CDK1 in promoting RNA Pol III activity during cell cycle progression [26, 27]. Additionally, CDK1 has been shown to regulate respiratory activity by enhancing the importation of Tom 40 into the mitochondria during mitosis [47]. Mitochondrial hyperfusion is observed during the G1 to S transition, whereas mitochondrial hyperfission occurs during mitotic transition [48]. Previous studies showed that Drp1 is critical in regulating mitochondrial fission [49], and Aurora-A can promote the activation of Drp1 through the Cyclin-B-CDK1 pathway [50, 51]. In this study, our results indicated that Aurora-A can suppress the nuclear function of Maf1 and promote its cytosolic accumulation. Our unpublished results show that overexpressed Maf1 predominantly localizes to the cytosol and can promote mitochondrial fission in HCC cells. These findings suggest that the regulation of Aurora-A on Maf1 may play a crucial role during cell cycle progression, and this may contribute to tumorigenesis.

Previous studies indicated that the C-domain of Maf1 is involved in its subcellular localization and its function in suppressing RNA pol III and protein stability [17]. Our study found that Aurora-A can interact with the C-domain of Maf1. We found that mutation of the Maf1 Thr-212 residue abolishes its phosphorylation by Aurora-A and leads to dominant nuclear localization; the Maf1/T212A mutant also loses its ability to suppress tRNA biosynthesis. Further investigation is required to assess whether Aurora-A can directly phosphorylate the Thr-212 residue on Maf1. Overexpression of Aurora-A prolongs the protein stability of Maf1 in a kinase-independent manner. However, the protein stability is attenuated in Maf1/T212A mutant. These results suggest that the phosphorylation status of Maf1 Thr-212 residue may play a critical role in the Aurora-A-mediated enhancement of protein stability. Overexpression of Aurora-A also promotes the cytosolic accumulation of Maf1, suggesting a crucial role of cytosolic Maf1 in tumorigenesis.

Previous studies demonstrated that modification of mitochondrial tRNA^Met^ plays an important role in modulating mitochondrial function, and oral cancer cells deficient in this modification exhibit increased glycolysis [9]. In this study, we observed that Maf1 can localize to the mitochondria. Interestingly, the Maf1/T212A mutation abolished its dominant nuclear localization and slightly reduced the mitochondrial localization. This finding suggests that the phosphorylation of Maf1 may not be the key mediator in regulating the mitochondrial localization of Maf1. Additionally, the interaction between Aurora-A and Maf1/T212A mutant is slightly reduced compared to the interaction between Aurora-A and Maf1/wild type. This reduction level is similar to the attenuated mitochondrial localization of the Maf1/T212A mutant. Previous studies have shown that Aurora-A can translocate to the mitochondria and influence energy production [52, 53]. Our results suggest that Maf1 may localize to the mitochondria through its interaction with Aurora-A. Furthermore, overexpression of Maf1/WT, but not the Maf1/T212A mutant, influences SDHB expression and the ECAR level (Fig. 6). Overexpression of the Maf1/T212A mutant fails to suppress tRNA biosynthesis. Those results imply that Maf1 may impact mitochondrial function by affecting the regulation of mitochondrial tRNA. In conclusion, our study reveals Aurora-A as a novel regulator of Maf1, controlling its subcellular localization and protein stability. The cytosolic localization of Maf1 promotes cell proliferation by affecting mitochondrial function. For HCC patients with high expression of both Aurora-A and Maf1, targeting Aurora-A may be a promising strategy in cancer therapy.

## Materials and methods

### cDNA constructs

The *Maf1* cDNA was amplified from breast cancer cell lines and cloned into the pcDNA3-HA vector. The pcDNA3-HA-*Maf1*/WT, pcDNA3-HA-*Maf1*/A, pcDNA3-HA-*Maf1*/BC, and pcDNA3-HA-*Maf1*/AB constructs were generated using TaKaRa Ex Taq® DNA Polymerase (PR001A, TAKARA). The T212A and S214A mutations in the pcDNA3-HA-*Maf1* construct were generated using the KAPA HiFi PCR Kit (KK2101, KAPA BIOSYSTEMS) according to the manufacturer’s instructions.

### Cell culture, transient transfection, and chemical treatment

Huh7 cells were obtained from the Japanese Collection of Research Bioresources Cell Bank (Osaka, Japan); Hep3B cells were obtained from the Bioresource Collection and Research Center (Taipei, Taiwan). Huh7 cells were cultured in DMEM/Low glucose medium (31600-034, GIBCO); Hep3B cells were cultured in DMEM/High glucose medium (12800-017, GIBCO). Cell lines used in this study were authenticated by short tandem repeat (STR) profiling to confirm their identity (NCKU Center for Genomic Medicine, Tainan, Taiwan); mycoplasma contamination tests were routinely performed monthly to ensure cell culture integrity.

The culture media were supplemented with 10% fetal bovine serum (VW-8510-186, Avantor) and 1% Penicillin-Streptomycin (30-02-Cl, CORNING). All cells were maintained at 37 °C and 5% CO_2_. Plasmids were transiently transfected into Huh7 and Hep3B cells using PolyJet™ transfection reagent (SL100688, SignaGen). For cycloheximide treatment, after 24 h post-transfection, the cells were treated with 20 μg/ml cycloheximide at different time points (0–4 h). For MG132 treatment, cells were treated with 20 μM MG132 for 4 h.

### Lentivirus-mediated shRNA infection

Lentivirus-mediated shGFP and shAurora-A were purchased from the RNA Technology Platform and Gene Manipulation Core (Taipei, Taiwan). Hep3B cells were infected with lenti-shGFP or shAurora-A containing 8 μg/ml polybrene in a culture medium. After 24 h, the infected cells were selected with 1.5 μg/ml puromycin for an additional 72 h. Hep3B-infected cells were passaged at least once before subsequent functional assays.

### Reverse transcription-quantitative real-time polymerase chain reaction (RT-qPCR)

Total RNA was extracted using TRIsure^TM^ reagent (BIO38032, Bioline) following the manufacturer’s instructions. 1–2 µg of total RNA was reverse transcribed into cDNA using the High-Capacity cDNA Reverse Transcription Kit (4368814, Thermo Fisher Scientific Inc.). For qPCR, 10-100 ng of cDNA was used with either SYBR Green kit (1708880, BioRad) or TaqMan® Universal Master Mix II (4366597, Applied Biosystems). The sequences of primers are provided in Supplementary Tables S1 and S2.

### Preparation of cell extracts, cell fraction extraction, co-immunoprecipitation assay, and western blot analysis

Total cell lysates were extracted using RIPA lysis buffer (50 mM Tris-HCl/pH 8.0, 150 mM NaCl, 0.5% sodium deoxycholate, 1% Nonidet P-40, 0.1% SDS, 1 mM DTT, 10 mM β-glycerol phosphate, and 1 mM EGTA), supplemented with a protease inhibitor cocktail (P8340, Sigma). For calf-intestinal alkaline phosphatase (CIP) treatment, the total cell lysate was treated with 20 units of CIP at 37 °C for 1 h. For co-immunoprecipitation, the lysates were extracted using IP lysis buffer (50 mM Tris-HCl/pH 8.0, 120 mM NaCl, 5 mM EGTA, 0.05% Triton X-100, and 0.055% NP-40), supplemented with a protease inhibitor cocktail (P8340, Sigma). Co-immunoprecipitation was performed using anti-GFP (#632381, Clontech) or anti-HA (ab9110, Abcam) antibodies. Cytoplasmic and nuclear fractions were extracted using the Nuclear Extraction Kit (SK0001, Signosis) according to the manufacturer’s instructions. Mitochondria were extracted using the Cell Fractionation Kit-Standard (ab109719, Abcam) according to the manufacturer’s instructions. Western blot analysis was performed using the following antibodies: Anti-HA (ab9110, Abcam), anti-GFP (632381, Clontech), anti-FLAG (F7425, Sigma), anti-Maf1 (GTX106776, Genetex), anti-Aurora-A (A1231, Sigma), anti-phospho-Aurora-A antibodies (3079, Cell Signaling), anti-OXPHOS (ab140411, Abcam), anti-Lamin A/C (sc-7292, Santa Cruz), anti-VDAC (ab15895, Abcam), and anti-α-tubulin (T6199, Sigma). Full and uncropped western blot images are available in the Supplementary Materials.

### Immunofluorescence assay

Huh7 cells were seeded onto sterile coverslips and transfected with pcDNA3-HA-Maf1. Twenty-four hours post-transfection, the cells were fixed with 3.7% formaldehyde (P3813, Sigma) for 10 min, followed by permeabilization with 0.05% NP-40. Immunofluorescence staining was performed using an anti-HA antibody (ab9110, Abcam). The cells were counterstained with DAPI and MitoTracker Red to visualize the nuclei and mitochondria in accordance with the manufacturer’s instructions.

### In situ proximity ligation assay (PLA)

Huh7 and Hep3B cells were seeded on a sterile coverslip and transfected with pEGFP-Aurora-A and pcDNA3-HA-Maf1; after 24 h of transfection, cells were fixed with 3.7% formaldehyde (P3813, Sigma) for 10 min. In situ PLA assay (DUO92101, Sigma) was conducted according to the manufacturer’s instructions using anti-HA and anti-GFP antibodies. The interaction between HA-Maf1 and GFP-Aurora-A or endogenous Maf1 and Aurora-A was visualized as distinct bright-red spots and detected using a fluorescence microscope.

### Cell proliferation

Huh7 cells were transiently transfected with pcDNA3-HA, pcDNA3-HA-*Maf1*, pcDNA3-HA-*Maf1*/T212A, and pcDNA3-HA-*Maf1*/S214A. After 96 h of transfection, cell proliferation was assessed using the CCK-8 (CK04, DOFINSO). For Aurora-A inhibition, the cells were treated with MLN8237 (A10004, Adooq) at 24 h post-transfection, and the cell proliferation was evaluated using the CCK-8 at 48 h post-MLN8237 treatment.

### In-gel digestion of proteins

Gel slices were reduced with 200 μL of 20 mM dithiothreitol (DTT) at 55 °C for 30 min, followed by centrifugation at 15,000 rpm to remove the supernatant. Alkylation was performed with 200 μL of 55 mM iodoacetamide (IAA) at room temperature for 60 min in the dark. After centrifugation at 15,000 rpm to discard the supernatant, the gel pieces were dried completely in a SpeedVac concentrator. Proteins were digested with sequencing-grade trypsin (Promega, enzyme-to-protein ratio of 1:20, w/w) at 37 °C for 16 h. Peptides were subsequently extracted from the gel with 100–200 μL of 5% formic acid in 50% acetonitrile, and the combined extracts were dried in a SpeedVac before LC-MS/MS analysis.

### LC-MS/MS analysis and database search

LC–MS/MS analysis was performed on an Orbitrap Fusion Lumos Tribrid quadrupole-ion trap-Orbitrap mass spectrometer (Thermo Fisher Scientific, San Jose, CA, USA) equipped with a NanoSpray ion source. Peptides were separated using an Ultimate 3000 nanoLC system (Thermo Fisher Scientific, Bremen, Germany). Peptide mixtures were loaded onto a C18 Acclaim PepMap NanoLC column (75 μm ID × 25 cm, 2 μm particle size, 100 Å pore size; Thermo Fisher Scientific, San Jose, CA, USA). Mobile phase A consisted of 0.1% formic acid in water, and mobile phase B consisted of 100% acetonitrile with 0.1% formic acid. Peptides were separated using a segmented 60-min gradient from 2% to 35% solvent B at a flow rate of 300 nL/min and a column temperature of 35 °C.

The mass spectrometer was operated in data-dependent acquisition (DDA) mode. Full MS scans were acquired at a resolution of 120,000 (m/z 200), externally calibrated to a mass accuracy of <5 ppm, with an Automatic Gain Control (AGC) target of 5 × 10⁵ and a maximum injection time of 50 ms. The most intense precursor ions within a 3-s cycle were selected for higher-energy collisional dissociation (HCD) fragmentation at normalized collision energies of 15, 30, and 45. MS/MS spectra were acquired at a resolution of 15,000 with a 1.4 Da isolation window, an AGC target of 5 × 10⁴, and a maximum injection time of 50 ms. To reveal the modified site, targeted LC-MS/MS experiments were then performed in which precursor ions of interest were focused.

Raw MS/MS data were searched against the UniProtKB/Swiss-Prot MAF1 protein database (Accession: Q9H063, downloaded on September 11, 2025) using Mascot and SEQUEST search engines implemented in Proteome Discoverer (version 2.5, Thermo Fisher Scientific). Search parameters were as follows: precursor mass tolerance, 10 ppm; fragment mass tolerance, 0.02 Da; enzyme specificity, trypsin, allowing up to two missed cleavages. Variable modifications included methionine oxidation, asparagine and glutamine deamidation, and phosphorylation of serine, threonine, and tyrosine; carbamidomethylation of cysteine was set as a fixed modification. Relative peptide quantification across samples was performed based on raw peptide abundances to calculate abundance ratios.

### Extracellular acidification Rate (ECAR) assay

Huh7 cells were transiently transfected with pcDNA3-HA, pcDNA3-HA-*Maf1*, and pcDNA3-HA-*Maf1*/T212A. The glycolytic activity was assessed at 72 h post-transient transfection using the Extracellular Acidification Rate kit (ab197244, Abcam).

### Ethics approval and consent to participate

HCC specimens were obtained from the National Cheng Kung University Hospital following the principles outlined in the Declaration of Helsinki. The experimental protocol was approved by the Institutional Review Board (IRB) of the National Cheng Kung University Hospital, Tainan, Taiwan (B-ER-104-245). Informed consent was waived as the research involved no identifiable human data and posed minimal risk to participants.

### Bioinformatics analysis

The expression profile of *Maf1* mRNA in TCGA cancer data was obtained from the FireBrowse website (http://firebrowse.org/). The clinical outcomes of HCC patients with high or low *Maf1* mRNA expression in tumors were analyzed using the Kaplan-Meier plotter (https://kmplot.com/analysis/).

### Statistical analysis

Statistical differences between experimental groups were evaluated using either an unpaired Student’s *t*test (one-tailed) or two-way analysis of variance (ANOVA), as appropriate. Statistical significance is indicated as follows: ****, *p* < 0.0001; ***, *p* < 0.001; **, *p* < 0.01; *, *p* < 0.05.

## Supplementary information

Supplementary Tabels

Supplementary Figures

Supplementary Figure Legends

Original Data

Three independent experiments of western blot

## Data Availability

Original data are available from the corresponding author upon request. Uncropped full-length Western blot images can be found in the Supplementary Materials. The data underlying this article are available in the article and in its online supplementary material.
