# Supplementary material for: Aurora-A-mediated cytosolic localization of Maf1 promotes cell proliferation via regulating mitochondrial function in HCC
Source: Cell Death Discov. 2025 Dec 3;11:561. doi: 10.1038/s41420-025-02885-z (PMC12717421; doi:10.1038/s41420-025-02885-z)
Supplement: Supplementary file 1 — Supplementary Tabels [file 41420_2025_2885_MOESM1_ESM.pdf]

**Table S1. Primer sequences for SYBR Green qPCR assay.**

| Gene | | Sequence |
|---|---|---|
| ***Maf1*** | Forward | 5'-TGCCCACATCATTGGCA GGATTG-3' |
| | Reverse | 5'-TGAGCGTGGCAATCAGGTAGAAGA-3' |
| ***AURKA*** | Forward | 5'-AATGCCCTGTCTTACTG TCATTC-3' |
| | Reverse | 5'-TCCAGAGATCCACCTTCTCATC-3' |
| ***ACTIN*** | Forward | 5'-CTGGACTTCGAGCAAG AGATG-3' |
| | Reverse | 5'-TGATGGAGTTGAAGGTAGTTTCG-3' |
| ***Pre-tRNA-Leucin*** | Forward | 5'- GTCAGGATGGCCGAGTGGTCT -3' |
| | Reverse | 5'- CCACGCCTCCATACGGAGAAC -3' |
| ***Pre-tRNA-iMethionine*** | Forward | 5'- CTGGGCCCATAACCCAGAG -3' |
| | Reverse | 5'- TGGTAGCAGAGGATGGTTTC -3' |

**Table S2. Primer sequences for TaqMan® qPCR assay.**

| Gene | | Sequence |
|---|---|---|
| ***Pre-tRNA-Cysteine*** | Forward | 5'- GGGYATAGCTCAGTGGTAGAGCATT -3' |
| | Reverse | 5'- GGGCACCYGGATTTGAACC -3' |
| | Probe | 5'- CAGATCAAGAGGTCCC -3' |
| ***Pre-tRNA-Methionine*** | Forward | 5'- CCTCTTAGCGCAGYGGGC -3' |
| | Reverse | 5'- TGCCCTCTCTGAGGCTYG -3' |
| | Probe | 5'- CGTCAGTCTCATAATC -3' |
