# Supplementary material for: Aurora-A-mediated cytosolic localization of Maf1 promotes cell proliferation via regulating mitochondrial function in HCC
Source: Cell Death Discov. 2025 Dec 3;11:561. doi: 10.1038/s41420-025-02885-z (PMC12717421; doi:10.1038/s41420-025-02885-z)

# Supplementary Figure S1.

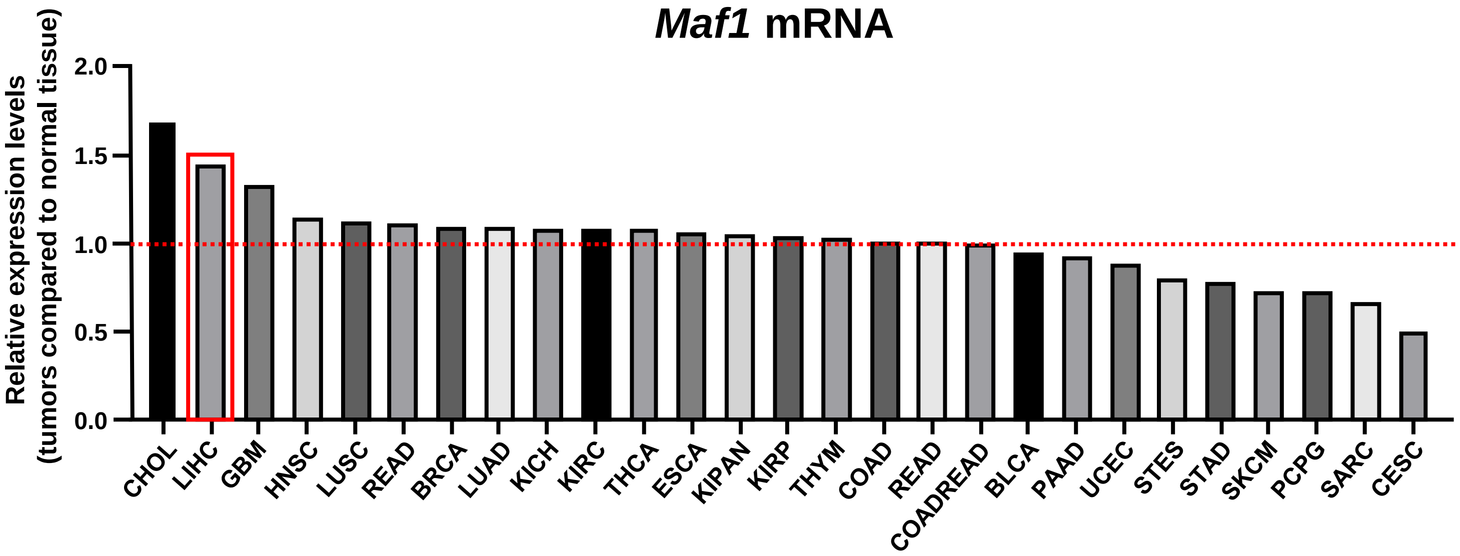

# Supplementary Figure S2.

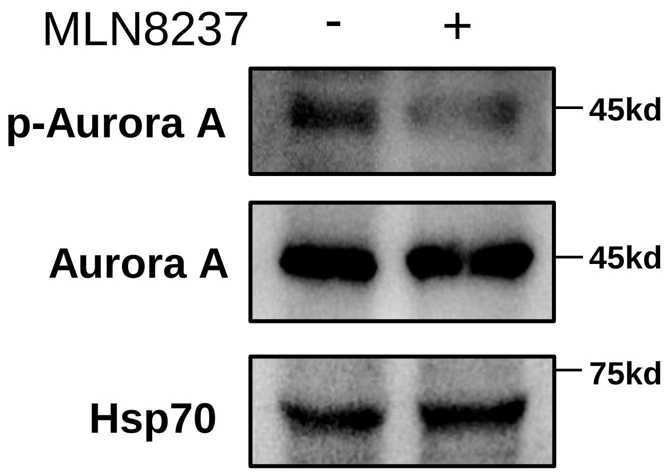

**Supplementary Figure S3.**

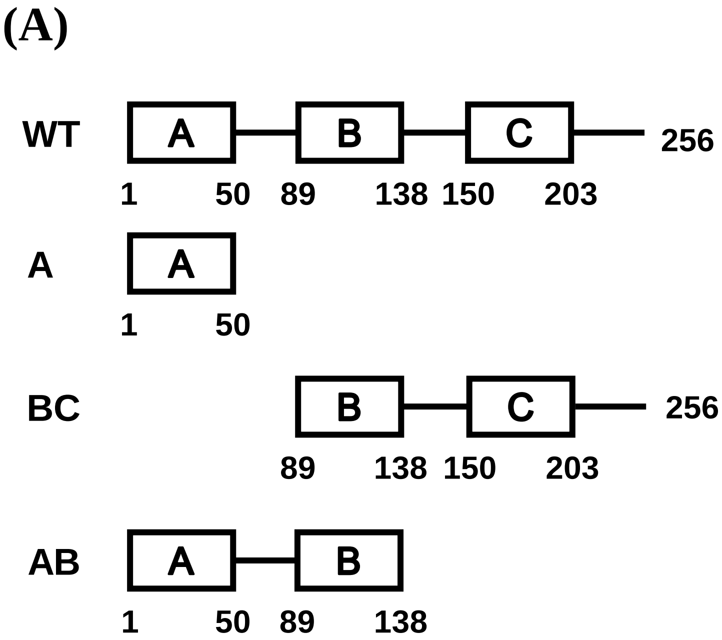


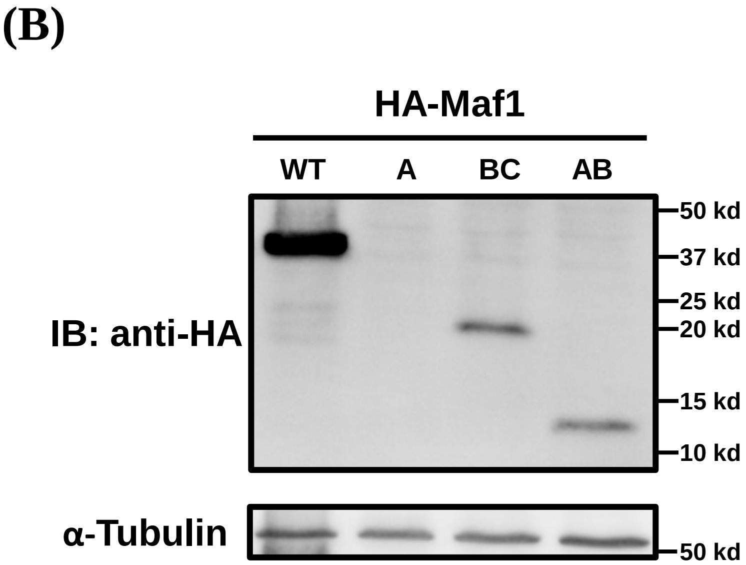

## Supplementary Figure S4.

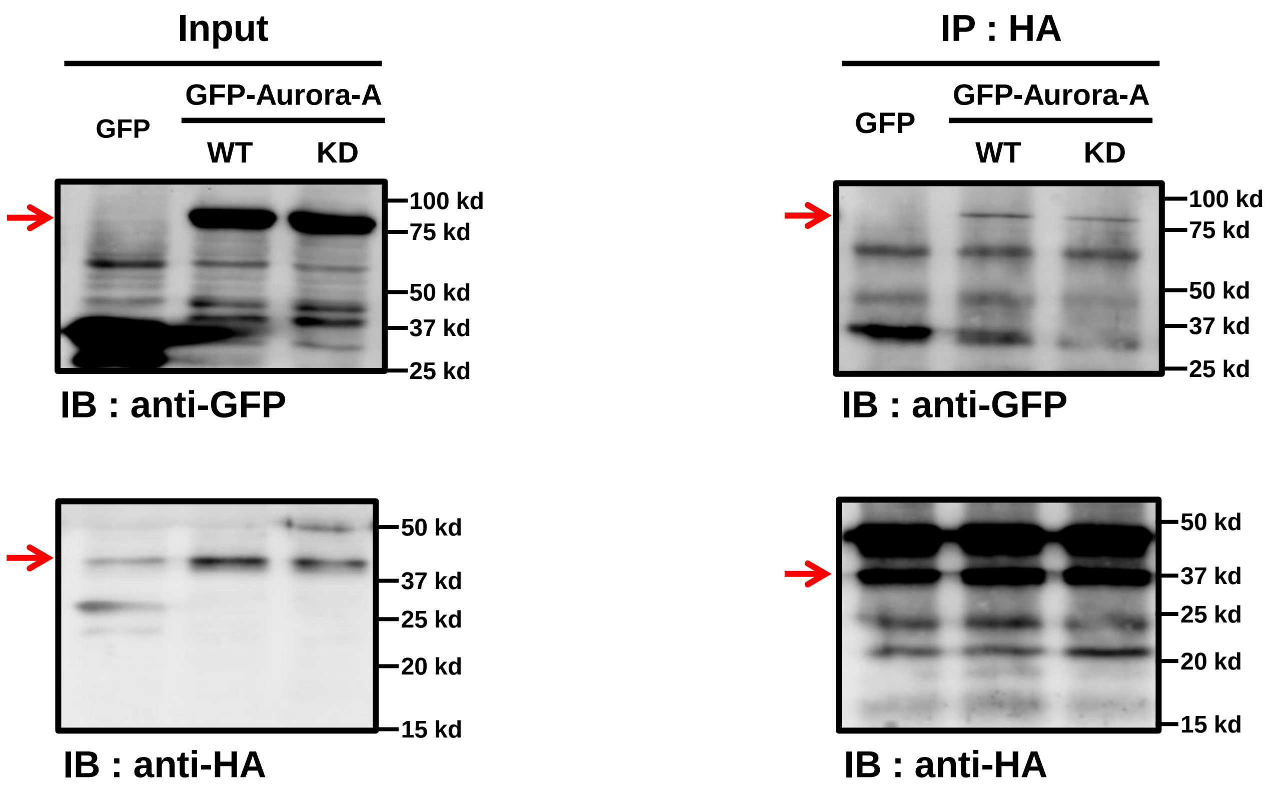

## Supplementary Figure S5.

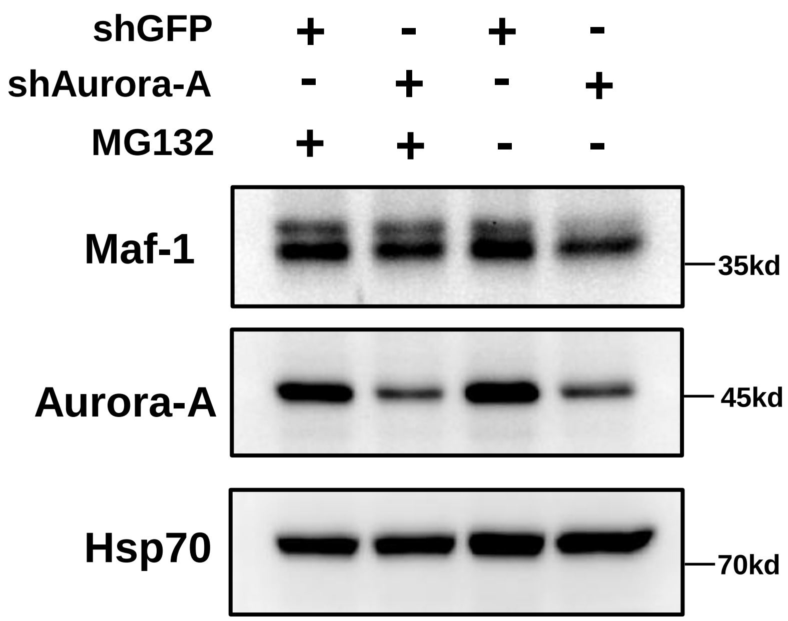

**Supplementary Figure S6.**

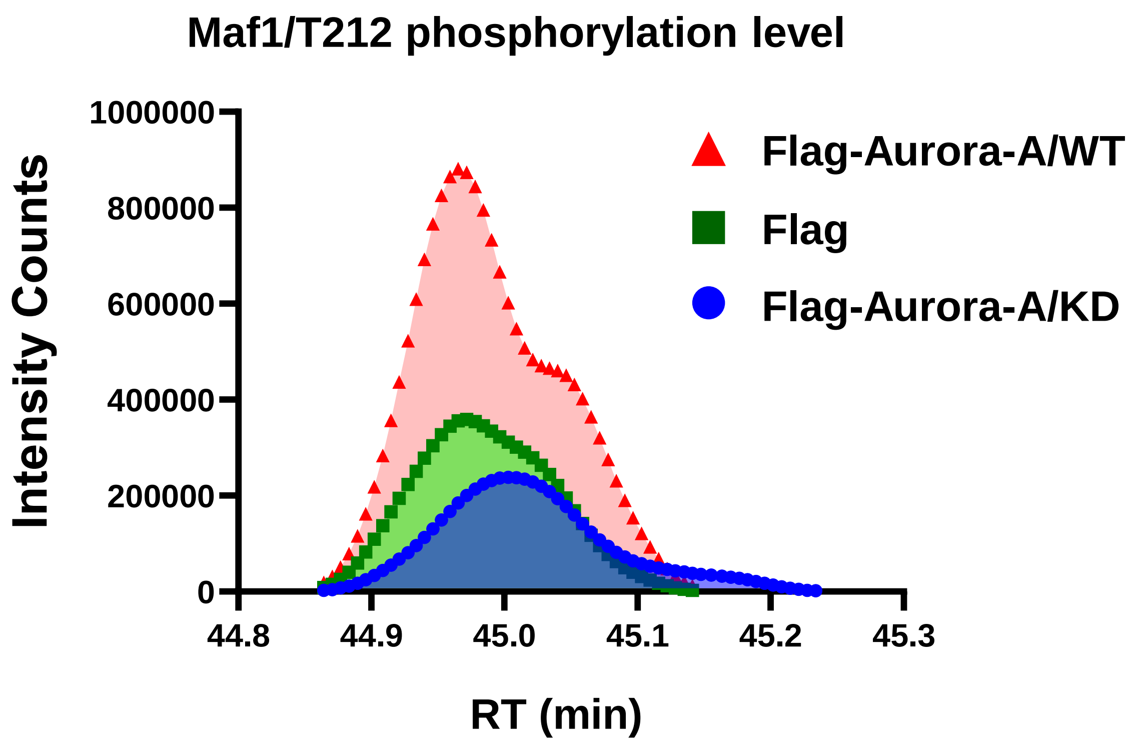

## Supplementary Figure S7.

**(A)**

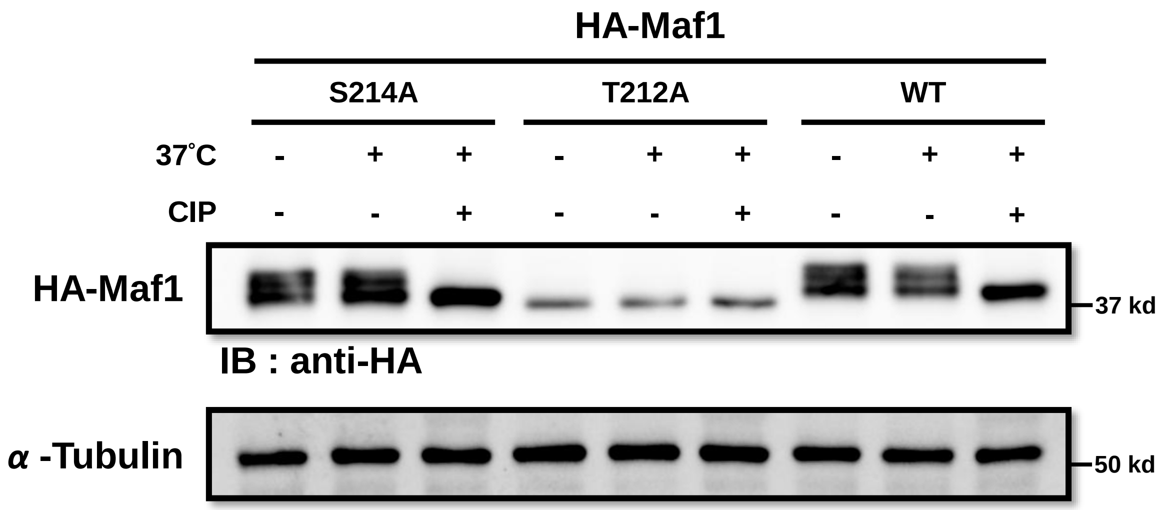


**(B)**

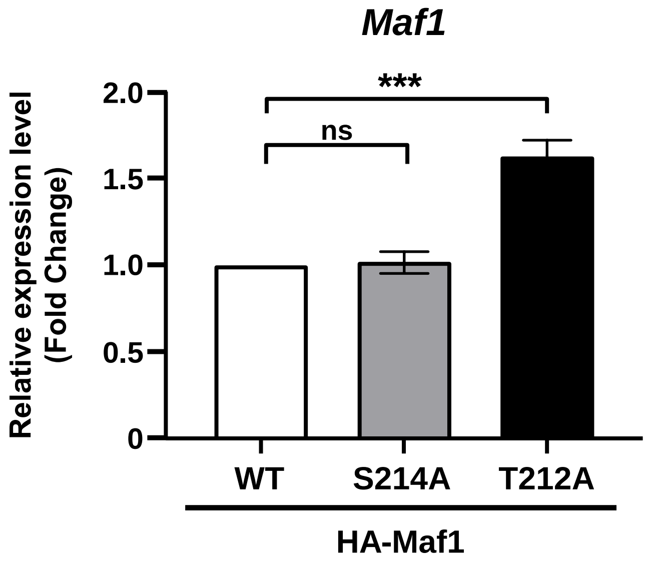

# Supplementary Figure S8.

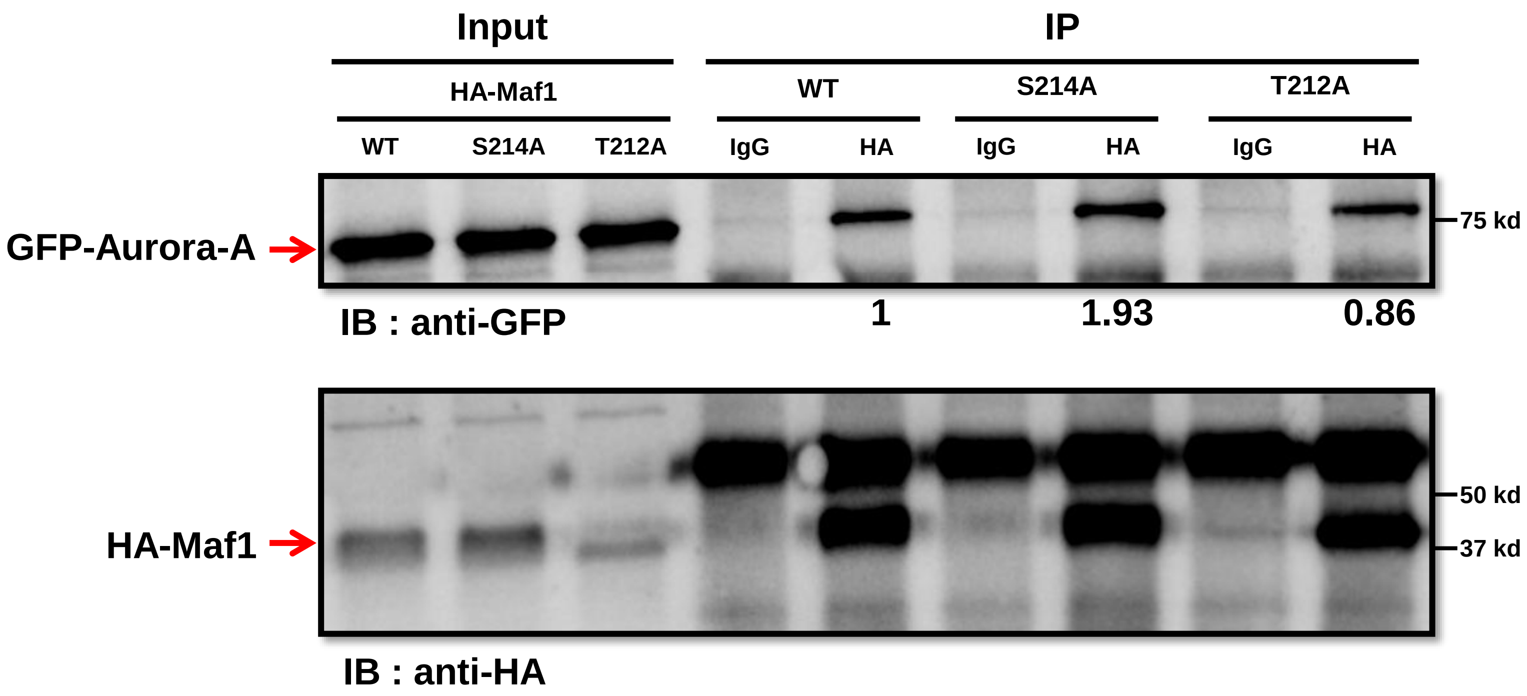

## Supplementary Figure S9.

(A)

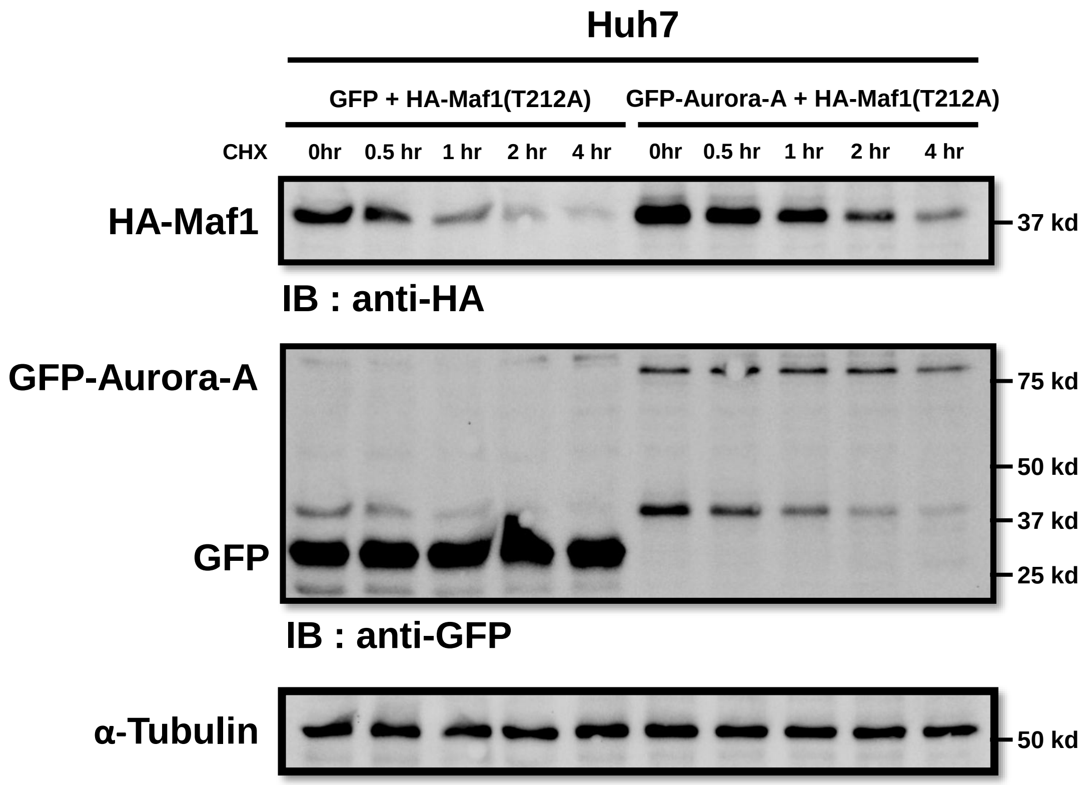


(B)

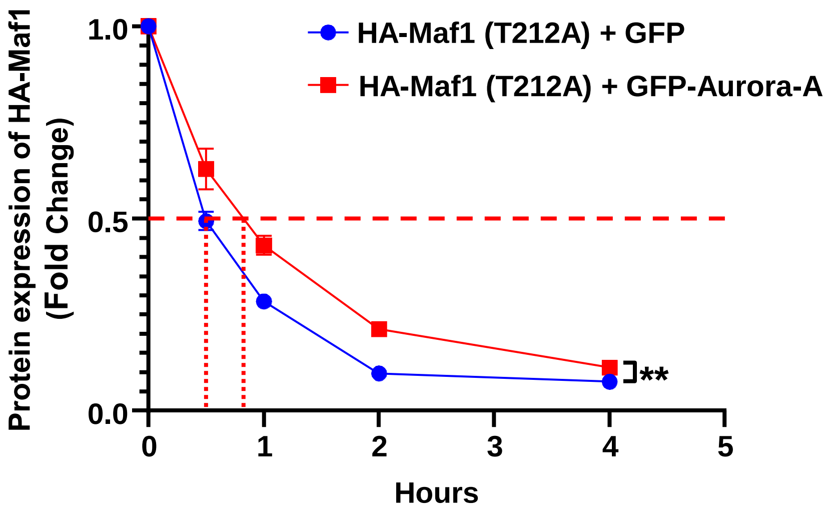


(C)

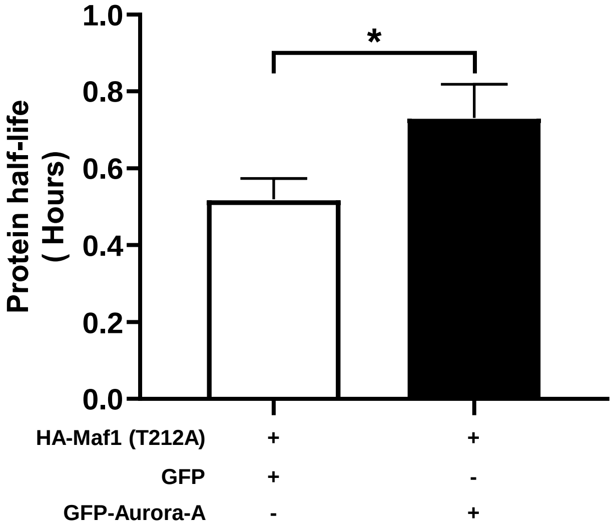

# Supplementary Figure S10.

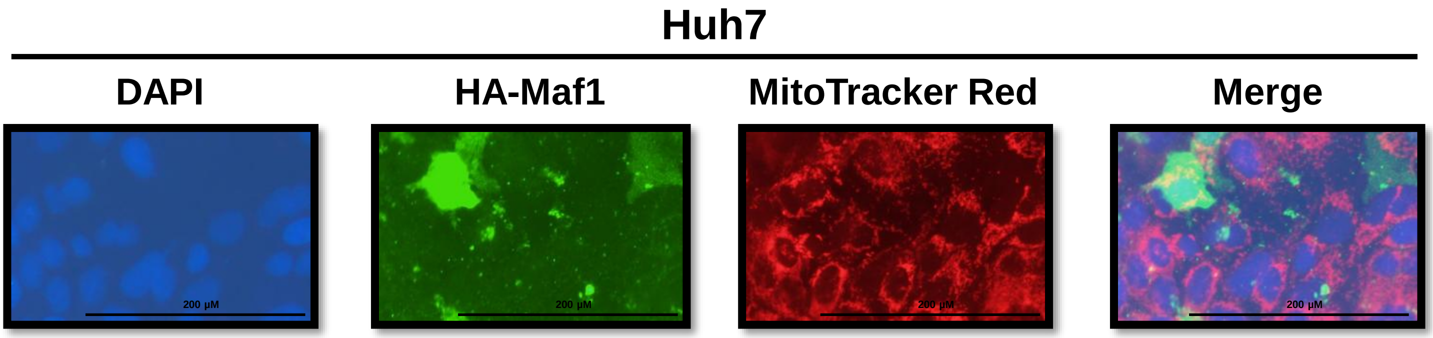

# Supplementary Figure S11.

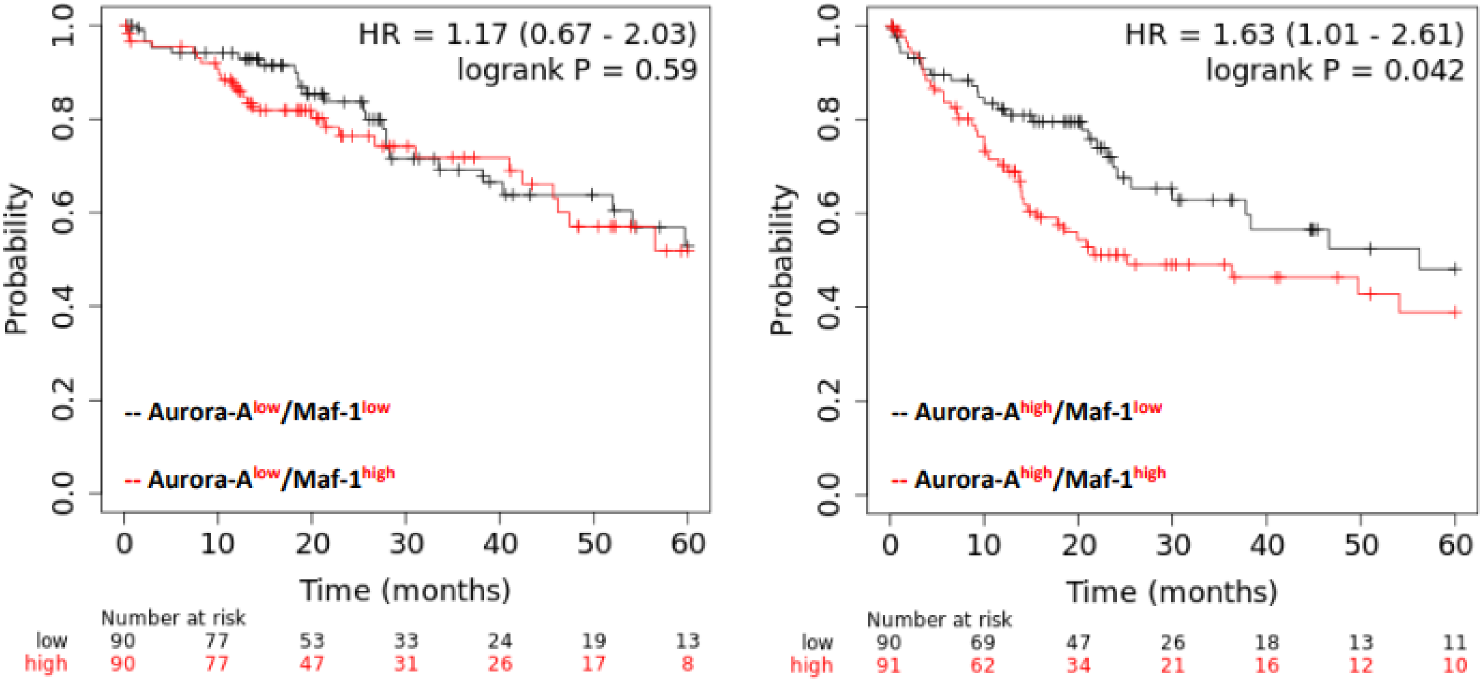

Supplement: Supplementary file 2 — Supplementary Figures [file 41420_2025_2885_MOESM2_ESM.pdf]
