# Supplementary material for: Aurora-A-mediated cytosolic localization of Maf1 promotes cell proliferation via regulating mitochondrial function in HCC
Source: Cell Death Discov. 2025 Dec 3;11:561. doi: 10.1038/s41420-025-02885-z (PMC12717421; doi:10.1038/s41420-025-02885-z)
Supplement: Supplementary file 3 — Supplementary Figure Legends [file 41420_2025_2885_MOESM3_ESM.pdf]

Supplementary Figure S1. ***Maf1*** **mRNA expression in various types of cancer in TCGA datasets.** The expression of *Maf1* mRNA in tumor tissues and adjacent normal tissues were retrieved from FireBrowse (http://firebrowse.org/). The mRNA expression values were presented as fold change in tumor tissues compared to their adjacent normal tissues.

Supplementary Figure S2. Inhibition of Aurora-A kinase activity by MLN8237 treatment. Hep3B cells were treated with (+) or without (-) 500 nM MLN8237 for 6 hours, and then the total cell lysates were harvested to perform western blot analysis using antibodies as indicated. Hsp70 is used as a loading control.

Supplementary Figure S3. Protein expression of Maf1 and its domain truncations. **(A)** Schematic diagram of Maf1 polypeptide with various truncations of BC-domain (A), A-domain (BC), and C-domain (AB). **(B)** The protein expression of each domain truncation was evaluated by western blot.

Supplementary Figure S4. Aurora-A kinase activity has a minimal impact on its interaction with Maf1. Huh7 cells were transiently co-transfected with HA-Maf1 and GFP or GFP-Aurora-A/WT or GFP-Aurora-A/KD. Total cell lysates were IB with indicated antibodies (left), and IP with anti-HA antibody (right) followed by IB with indicated antibodies. Red arrows indicate the expression of GFP-Aurora-A and HA-Maf1.

Supplementary Figure S5. Aurora-A regulates the protein stability of Maf1 via ubiquitin-dependent proteasome degradation. Hep3B cells infected with lenti-

shGFP or shAurora-A were treated with 20 μM MG132 (+) or not (-) for 4 hours. Total cell lysates were harvested for western blot analysis using antibodies as indicated. Hsp70 is used as a loading control.

Supplementary Figure S6. Aurora-A regulates the phosphorylation level of Maf1/T212. Hep3B cells were co-transfected with HA-Maf1 and Flag, Flag-Aurora-A/WT, or Flag-Aurora-A/KD. Total cell lysates were collected for immunoprecipitation assay using anti-HA antibodies and then subjected to liquid chromatography-tandem mass spectrometry (LC-MS/MS) analysis. The results were calculated from phosphopeptide abundances obtained by LC–MS/MS analysis. RT (retention time): minute; the abundance was shown as intensity counts.

Supplementary Figure S7. Thr-212 is the important residue for Maf1 phosphorylation. Huh7 cells were transiently transfected with HA-Maf1/WT, HA-Maf1/T212A, and HA-Maf1/S214A. **(A)** Total cell lysates were treated with (+) or without (-) calf intestinal alkaline phosphatase (CIP) at 37°C for 1 hour. Phosphorylated Maf1 (upper band) and unphosphorylated Maf1 (lower band) were detected by western blot using anti-HA antibody. α-Tubulin was used as a loading control. **(B)** The expression of *Maf1* mRNA was analyzed by RT-qPCR. Statistical analysis was performed using Student's t-test. ***, $p < 0.001$; ns, no significance.

Supplementary Figure S8. Maf1 phosphorylation has a minimal effect on its interaction with Aurora-A. Huh7 cells were transiently co-transfected with GFP-Aurora-A and HA-Maf1/WT, HA-Maf1/T212A, or HA-Maf1/S214A. Total cell lysates were collected to perform IP using anti-HA antibody, followed by IB using antibodies

as indicated. IgG was used as a negative control for IP. The intensities of the interaction between GFP-Aurora-A and HA-Maf1 were quantified.

Supplementary Figure S9. Aurora-A enhances the protein stability of Maf1/T212A mutant. Huh7 cells were transiently co-transfected with HA-Maf1/T212A and GFP (left) or GFP-Aurora-A. At 24 hours of post-transfection, the cells were treated with cycloheximide for various time periods (0-4 hours). **(A)** Cell lysates were collected to perform western blot using anti-HA and anti-GFP antibodies. α-Tubulin was used as a loading control. **(B)** Quantitative results of (A) were shown. The relative expression level of HA-Maf1 at each time point was compared to the 0-hour time point. Statistical analysis was performed using Two-way ANOVA. **, $p < 0.01$. **(C)** The protein half-life of the HA-Maf protein (from A) was shown. Student's t-test. *, $p < 0.05$.

Supplementary Figure S10. Maf1 localizes to the mitochondria in HCC cells. Huh7 cells were transiently transfected with HA-Maf1, and immunofluorescence analysis was performed using anti-HA antibodies to determine its subcellular localization (green). Mitochondria are labeled using MitoTracker (red). DNA is stained with DAPI (blue).

Supplementary Figure S11. Elevated expression of Maf1 and Aurora-A correlates with poor prognosis in HCC patients. Tumor tissues from HCC patients were stratified based on low (left) or high (right) *Aurora-A* mRNA expression and further categorized into low (black) or high (red) *Maf1* mRNA expression. Kaplan-Meier Plotter (https://kmplot.com/analysis/) was used to analyze the overall survival rate.
