# Supplementary material for: Aurora-A-mediated cytosolic localization of Maf1 promotes cell proliferation via regulating mitochondrial function in HCC
Source: Cell Death Discov. 2025 Dec 3;11:561. doi: 10.1038/s41420-025-02885-z (PMC12717421; doi:10.1038/s41420-025-02885-z)

Figure 2.

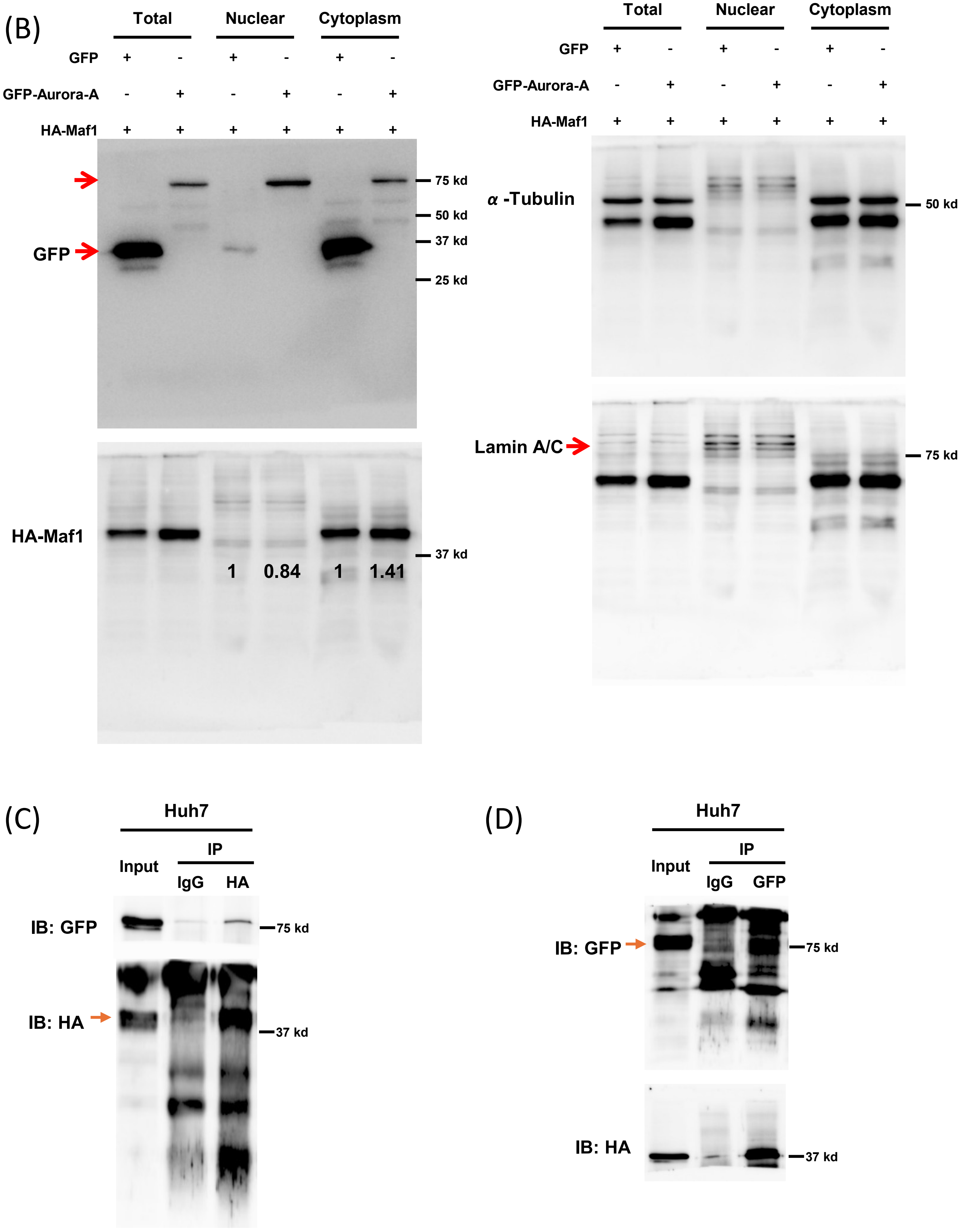

(B)
Total
Nuclear
Cytoplasm
GFP + - + - + -
GFP-Aurora-A - + - + - +
HA-Maf1 + + + + + +
GFP
75 kd
50 kd
37 kd
25 kd
HA-Maf1
1 0.84 1 1.41
37 kd
α -Tubulin
50 kd
Lamin A/C
75 kd
(C)
Huh7
Input
IP
IgG
HA
IB: GFP
IB: HA
75 kd
37 kd
(D)
Huh7
Input
IP
IgG
GFP
IB: GFP
IB: HA
75 kd
37 kd

Figure 2.

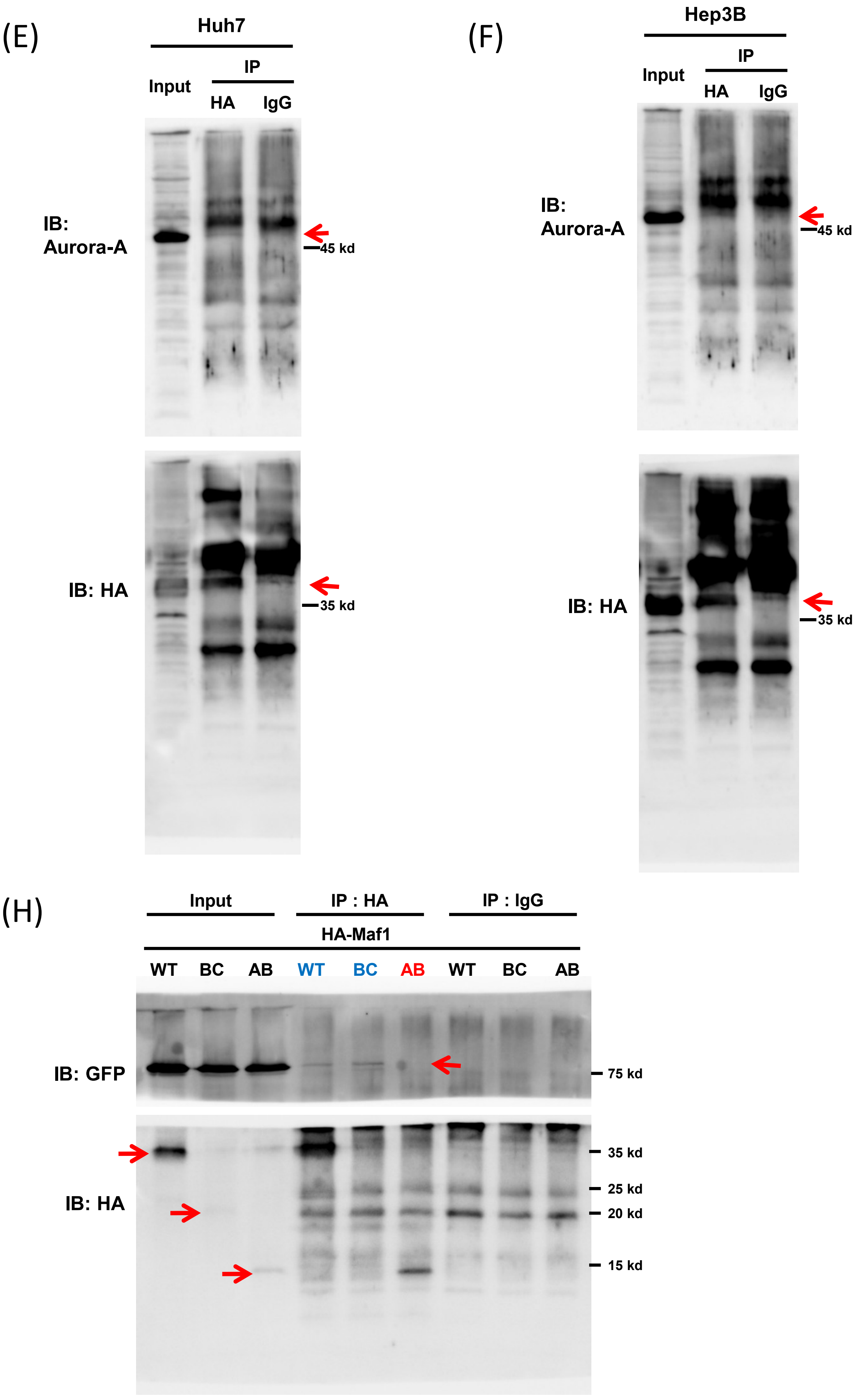

(E)
Huh7
IP
Input
HA
IgG
IB: Aurora-A
45 kd
IB: HA
35 kd
(F)
Hep3B
IP
Input
HA
IgG
IB: Aurora-A
45 kd
IB: HA
35 kd
(H)
Input
IP : HA
IP : IgG
HA-Maf1
WT BC AB WT BC AB WT BC AB
IB: GFP
75 kd
IB: HA
35 kd
25 kd
20 kd
15 kd

Figure 3.

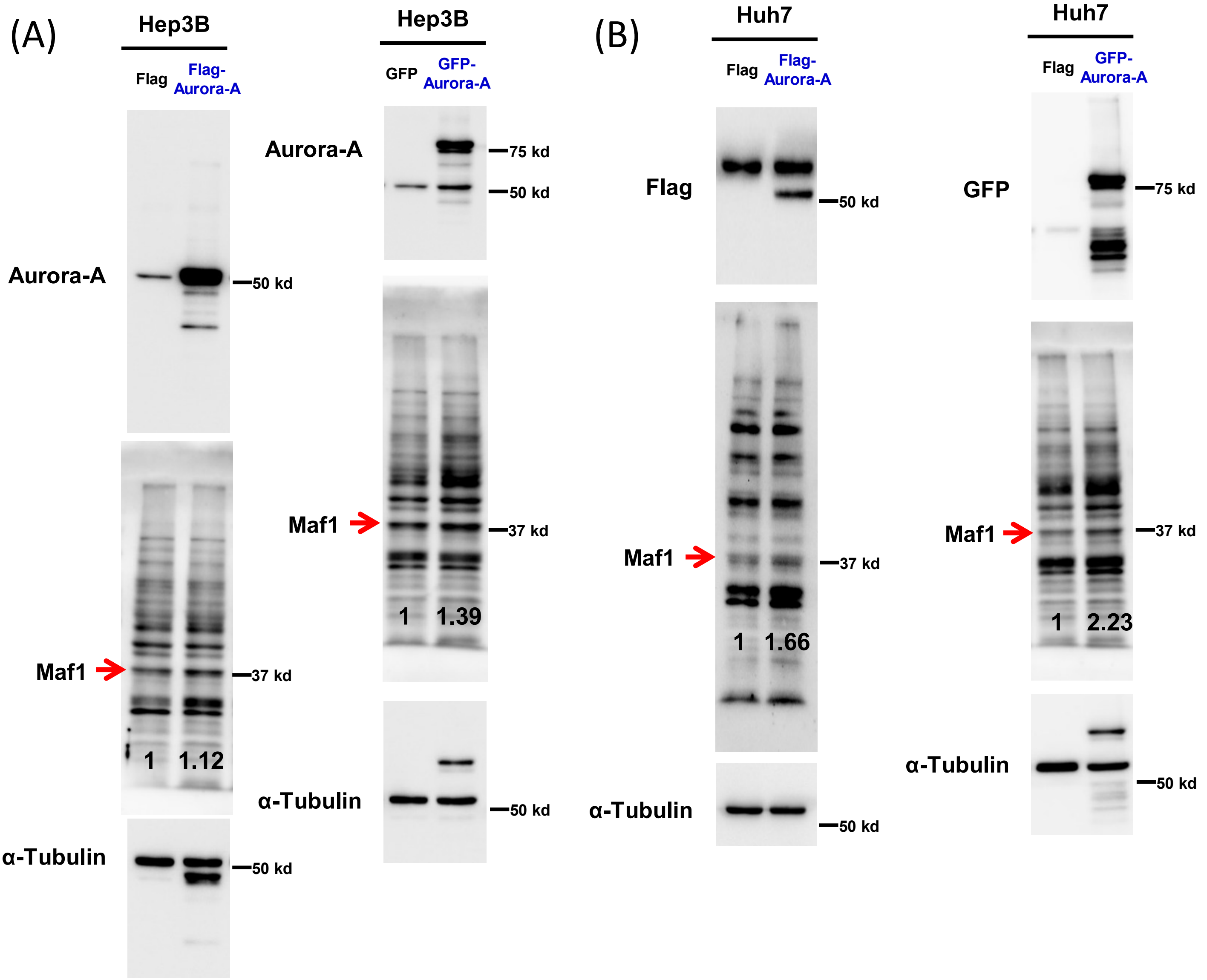

(A)
Hep3B
Flag
Flag-Aurora-A
Aurora-A
50 kd
Maf1
37 kd
1
1.12
α-Tubulin
50 kd
Hep3B
GFP
GFP-Aurora-A
Aurora-A
75 kd
50 kd
Maf1
37 kd
1
1.39
α-Tubulin
50 kd
(B)
Huh7
Flag
Flag-Aurora-A
Flag
50 kd
Maf1
37 kd
1
1.66
α-Tubulin
50 kd
Huh7
Flag
GFP-Aurora-A
GFP
75 kd
Maf1
37 kd
1
2.23
α-Tubulin
50 kd

Figure 3.

(D)

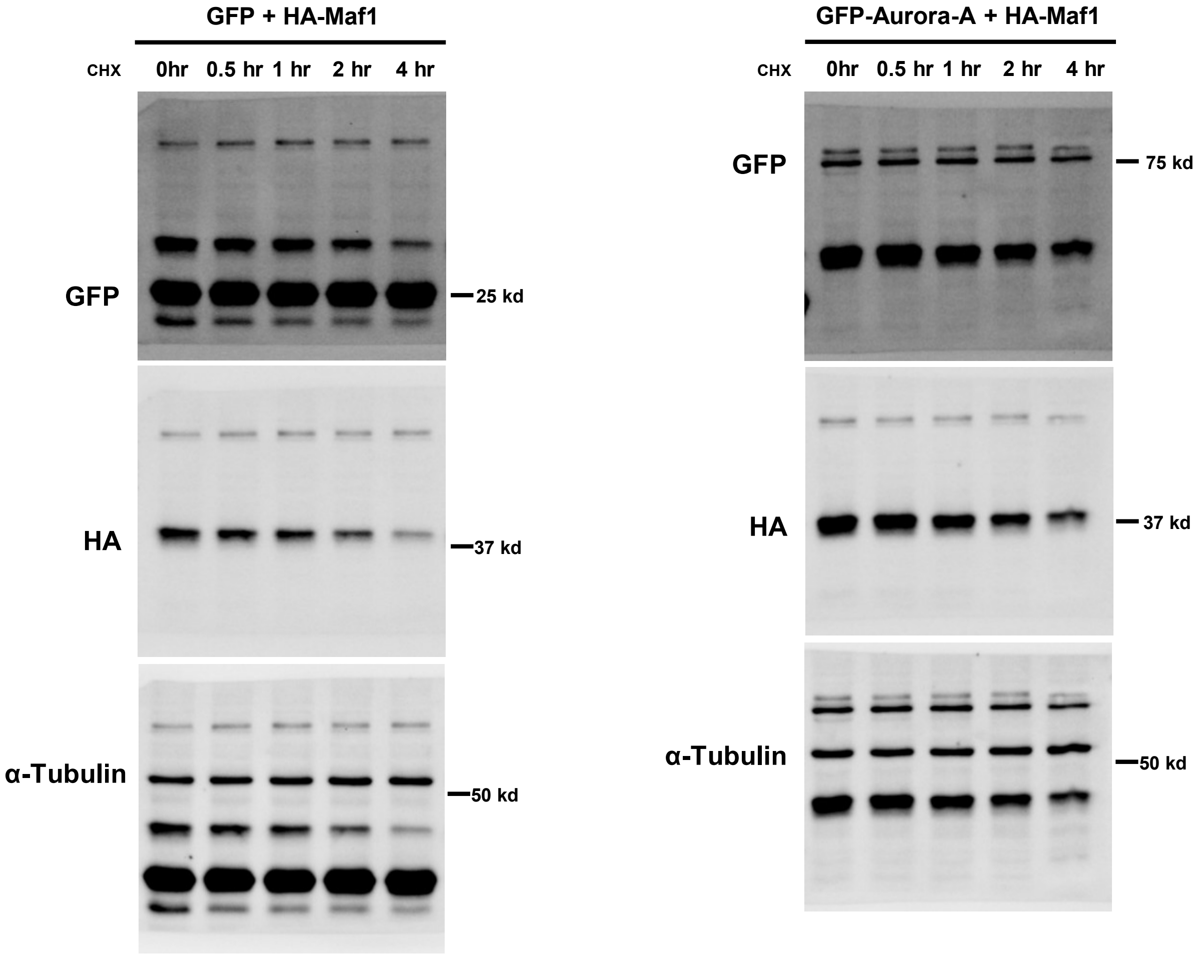
GFP + HA-Maf1
CHX
0hr
0.5 hr
1 hr
2 hr
4 hr
GFP
25 kd
HA
37 kd
α-Tubulin
50 kd
GFP-Aurora-A + HA-Maf1
CHX
0hr
0.5 hr
1 hr
2 hr
4 hr
GFP
75 kd
HA
37 kd
α-Tubulin
50 kd

Figure 3.

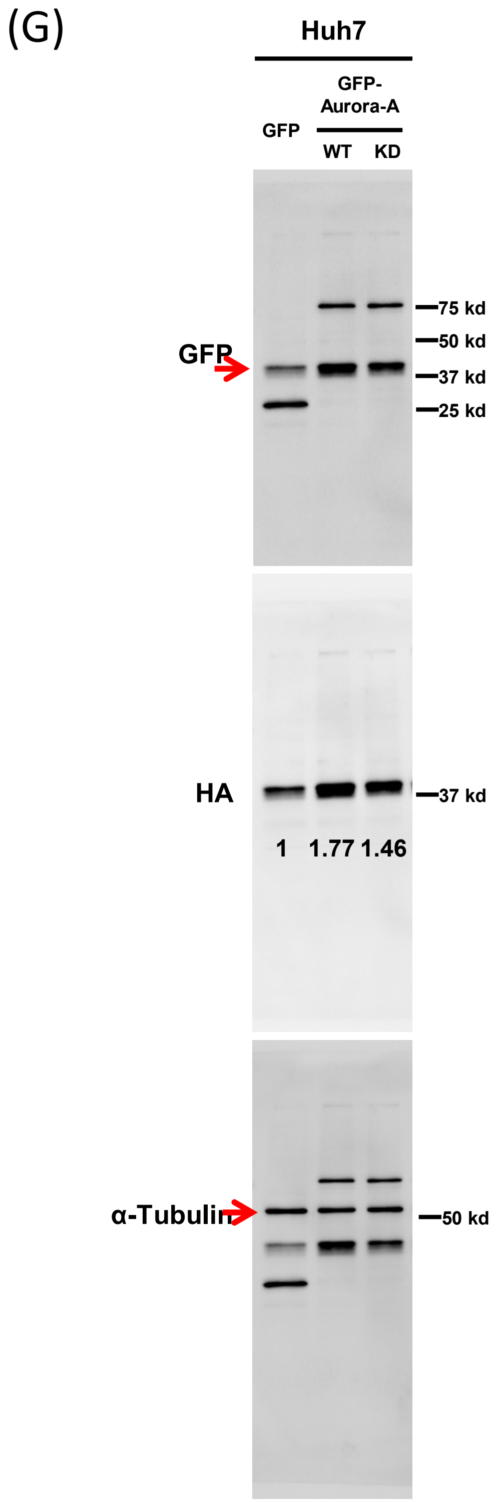

(G)
Huh7
GFP
GFP-Aurora-A
WT
KD
GFP
75 kd
50 kd
37 kd
25 kd
HA
37 kd
1 1.77 1.46
α-Tubulin
50 kd

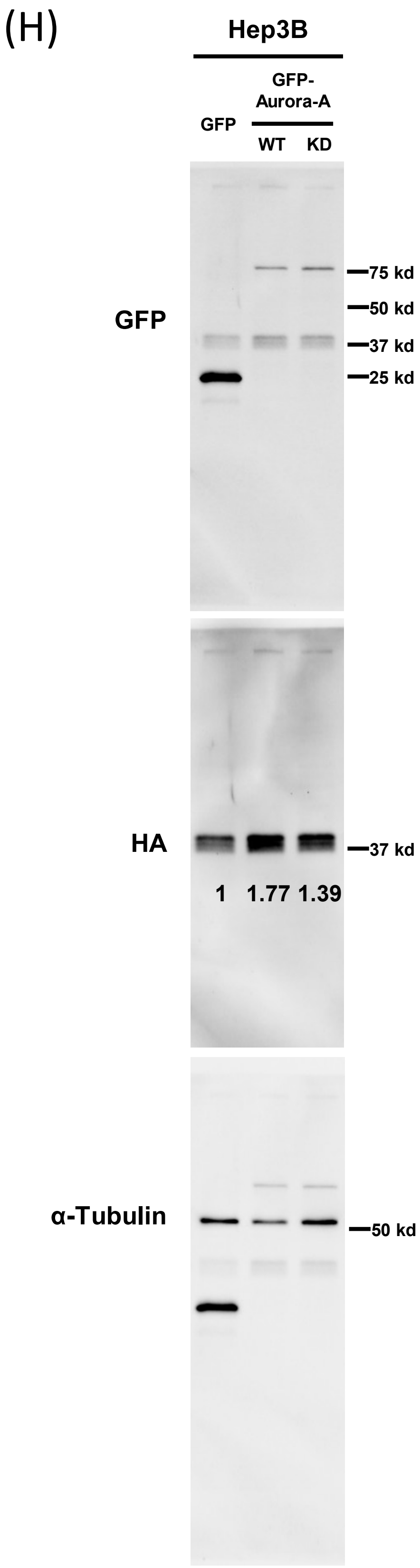

(H)
Hep3B
GFP
GFP-Aurora-A
WT
KD
GFP
75 kd
50 kd
37 kd
25 kd
HA
37 kd
1 1.77 1.39
α-Tubulin
50 kd

Figure 4.

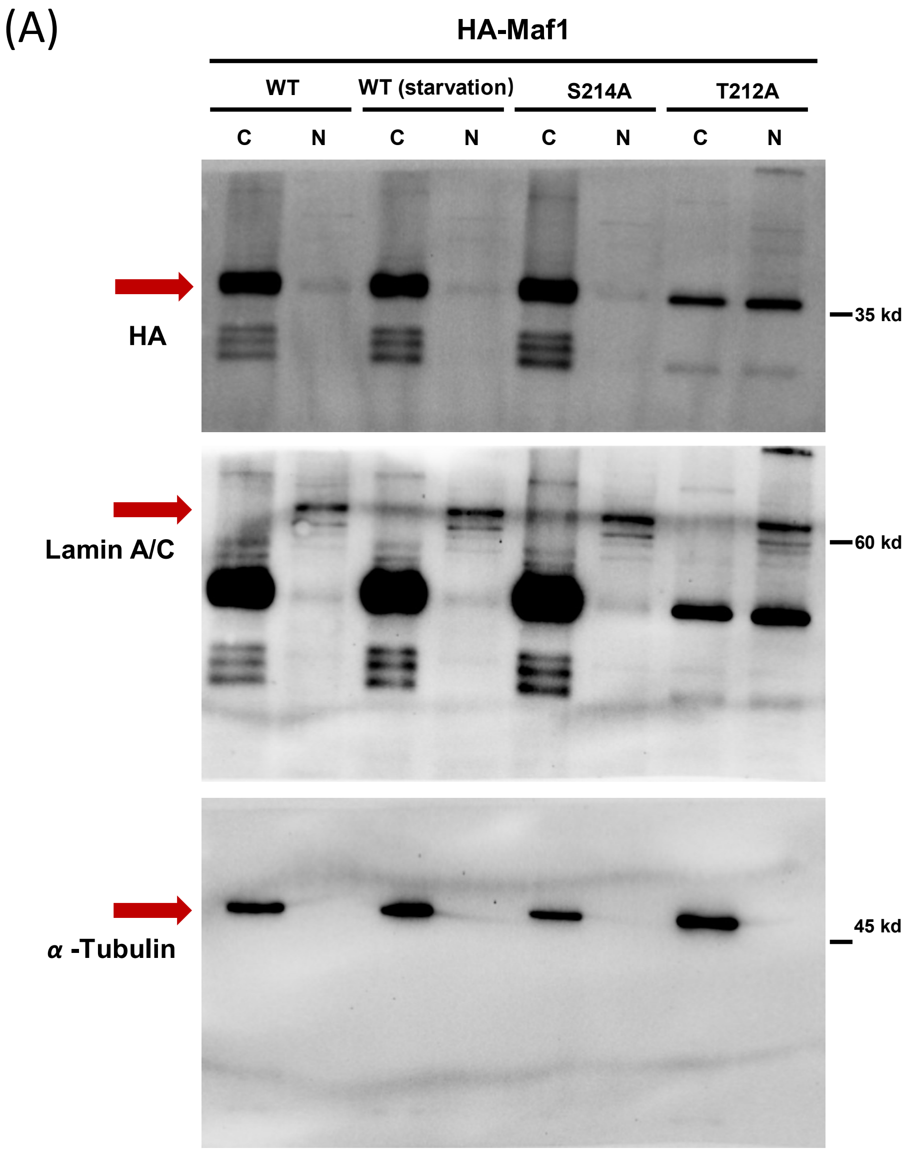
(A)
HA-Maf1
WT
WT (starvation)
S214A
T212A
C
N
C
N
C
N
C
N
HA
35 kd
Lamin A/C
60 kd
α -Tubulin
45 kd

Figure 4.

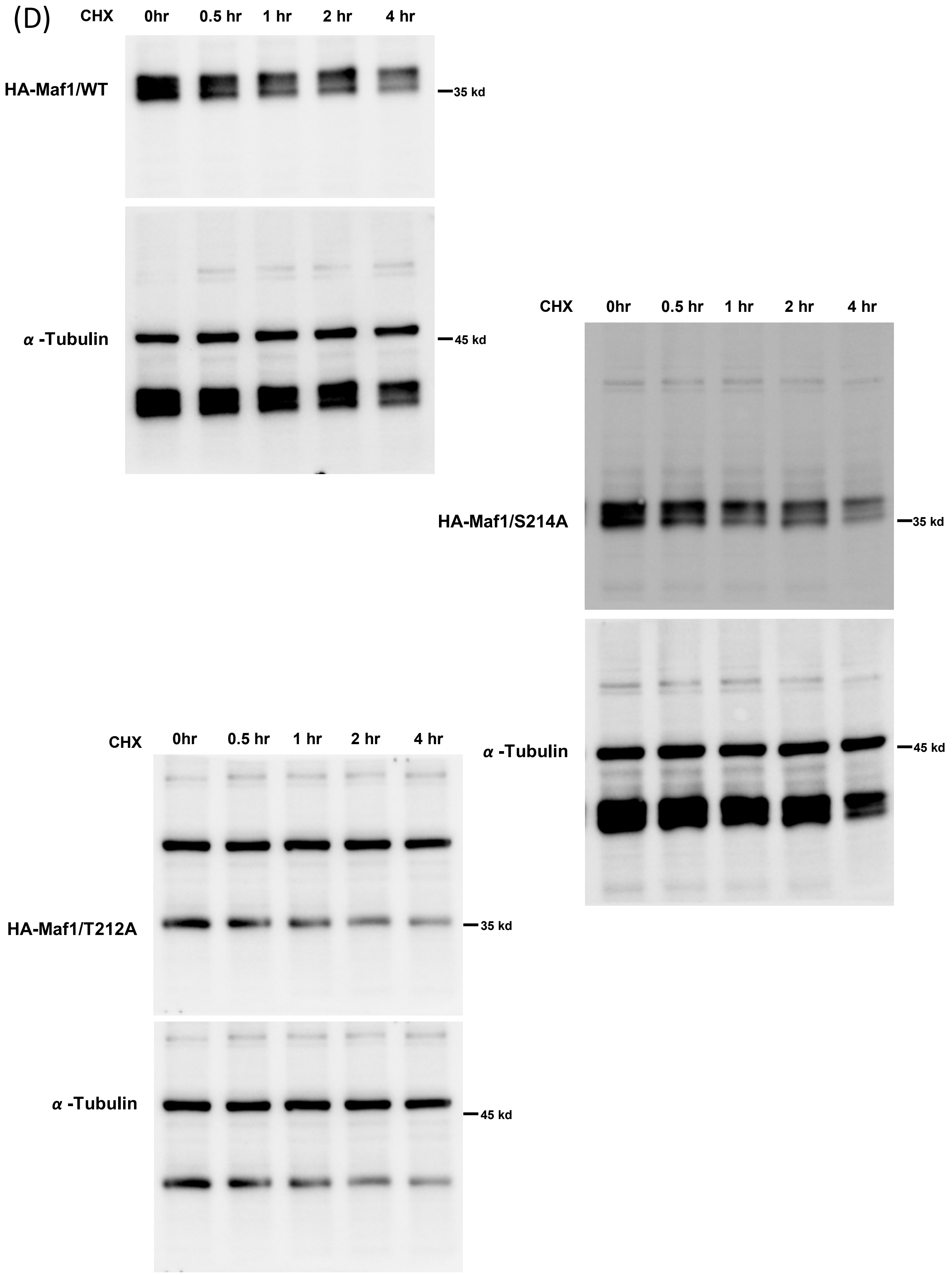
(D)
CHX
0hr
0.5 hr
1 hr
2 hr
4 hr
HA-Maf1/WT
35 kd
α -Tubulin
45 kd
CHX
0hr
0.5 hr
1 hr
2 hr
4 hr
HA-Maf1/S214A
35 kd
α -Tubulin
45 kd
CHX
0hr
0.5 hr
1 hr
2 hr
4 hr
HA-Maf1/T212A
35 kd
α -Tubulin
45 kd

Figure 4.

(F)

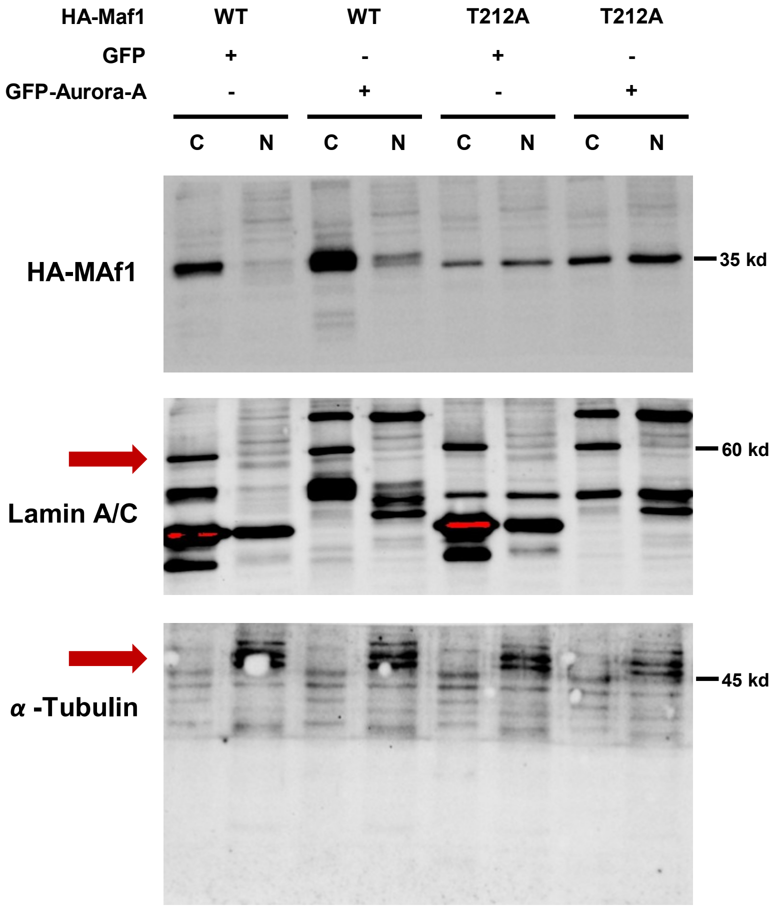

HA-Maf1
WT
WT
T212A
T212A
GFP
+
-
+
-
GFP-Aurora-A
-
+
-
+
C
N
C
N
C
N
C
N
HA-MAf1
35 kd
Lamin A/C
60 kd
α -Tubulin
45 kd

Figure 5.

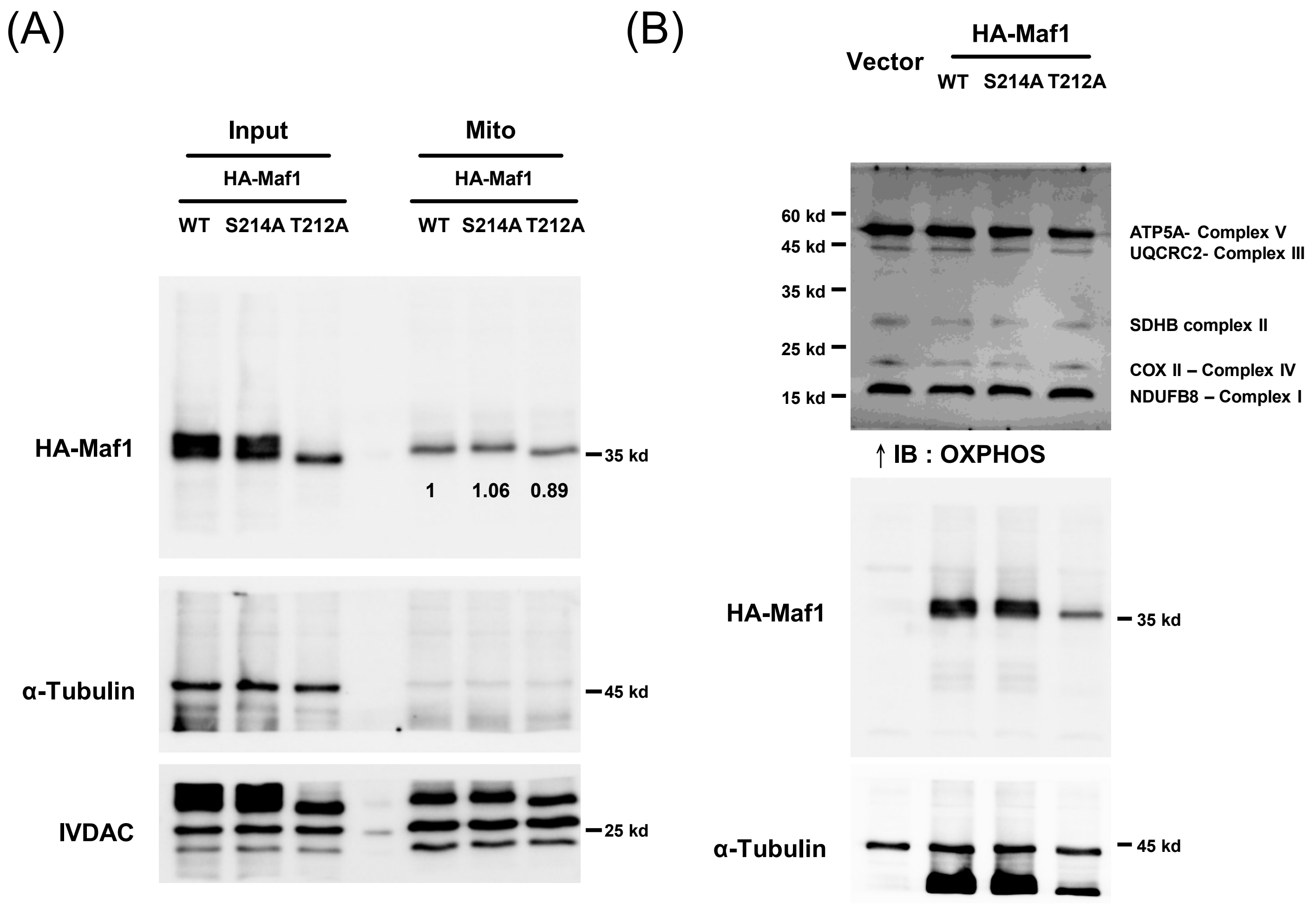

(A)
Input
HA-Maf1
WT S214A T212A
Mito
HA-Maf1
WT S214A T212A
HA-Maf1
35 kd
1 1.06 0.89
α-Tubulin
45 kd
IVDAC
25 kd
(B)
Vector
HA-Maf1
WT S214A T212A
60 kd
45 kd
35 kd
25 kd
15 kd
ATP5A- Complex V
UQCRC2- Complex III
SDHB complex II
COX II – Complex IV
NDUFB8 – Complex I
↑ IB : OXPHOS
HA-Maf1
35 kd
α-Tubulin
45 kd

Supplementary Figure S2.

(B)

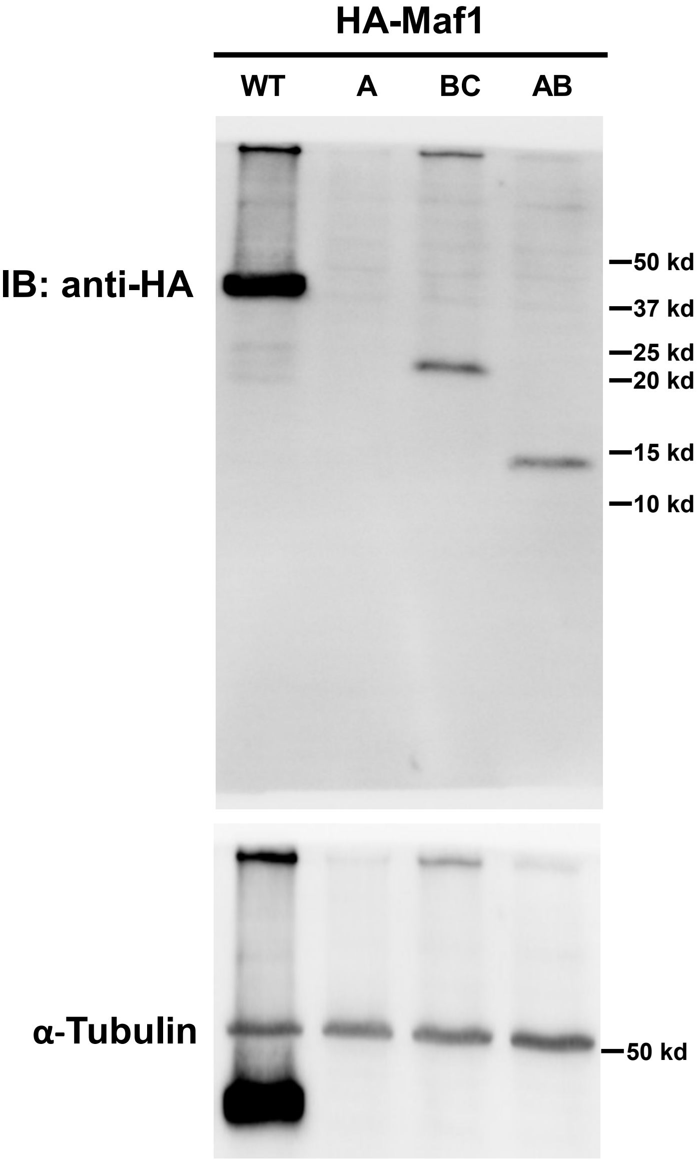

Supplementary Figure S3.

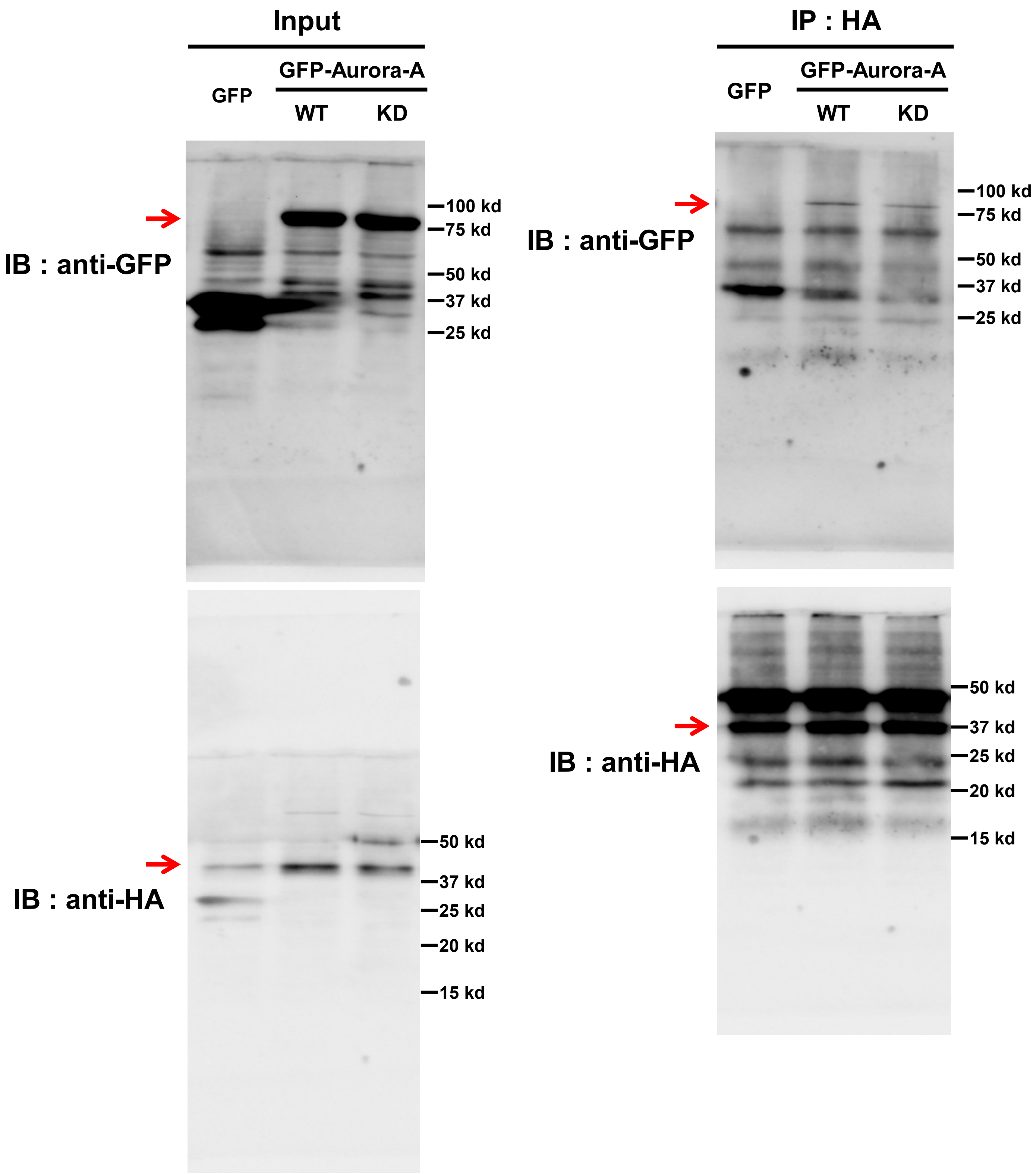

Supplementary S4.

(A)

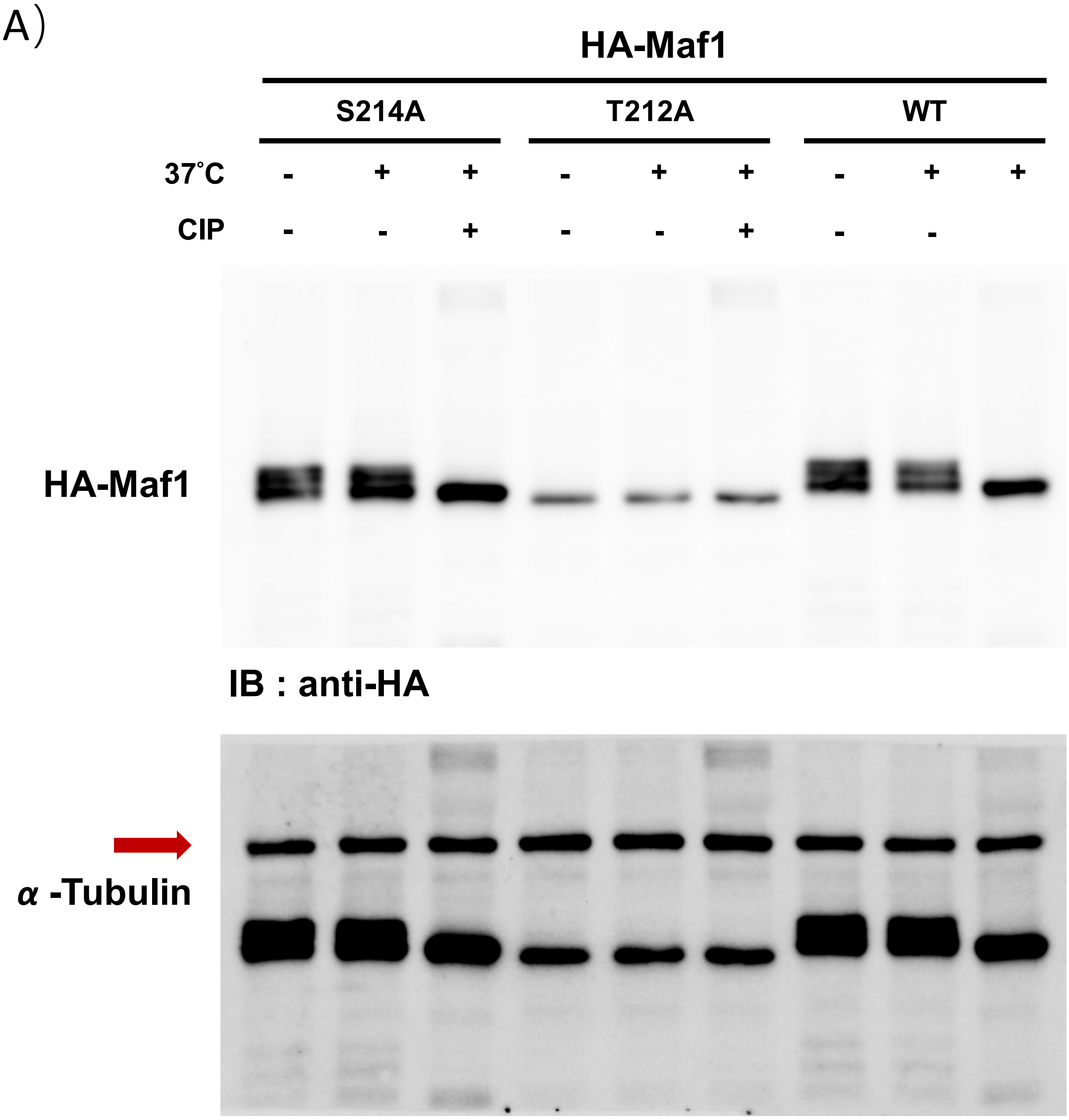

Supplementary S5.

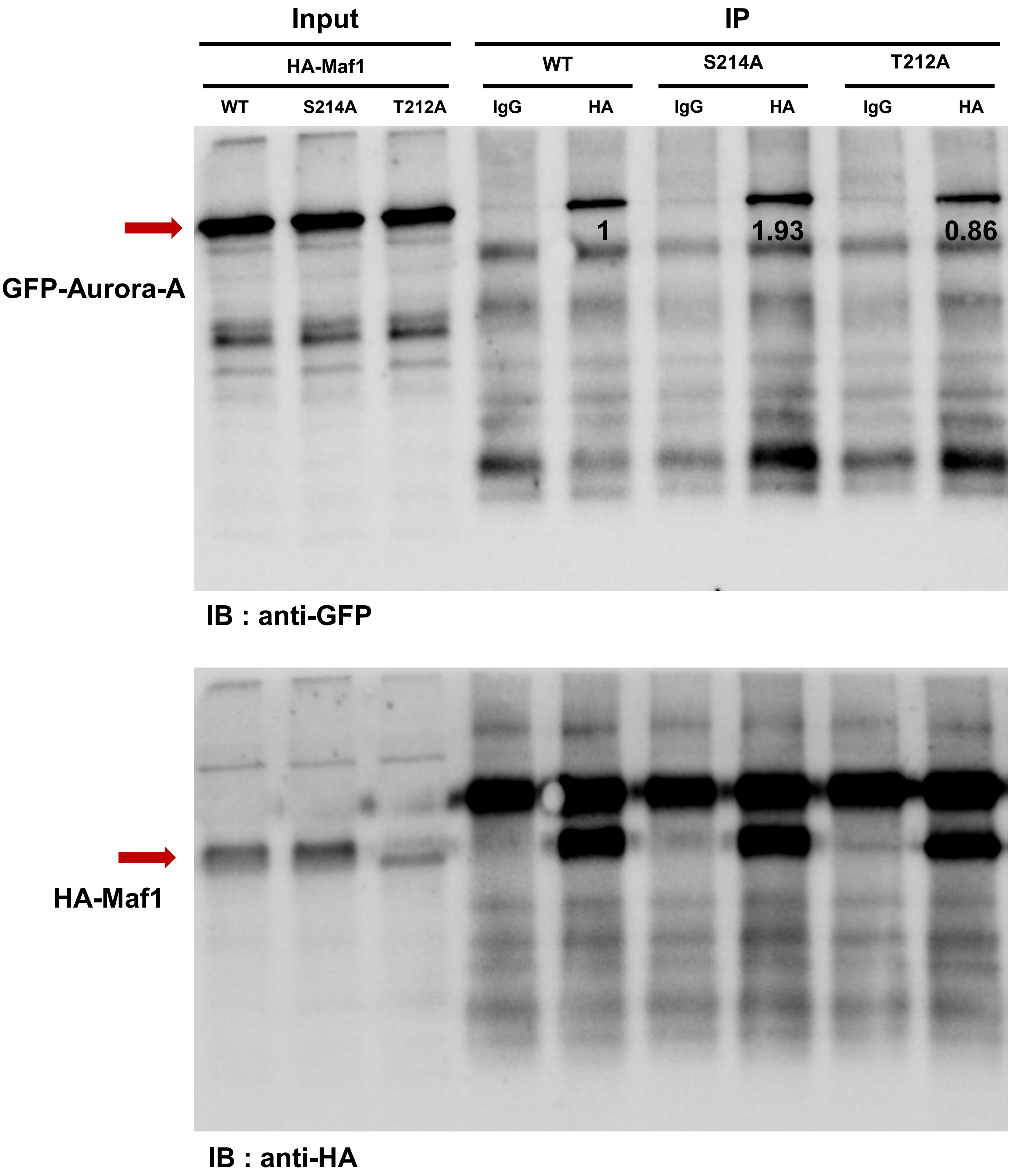

Supplementary S6.

(A)

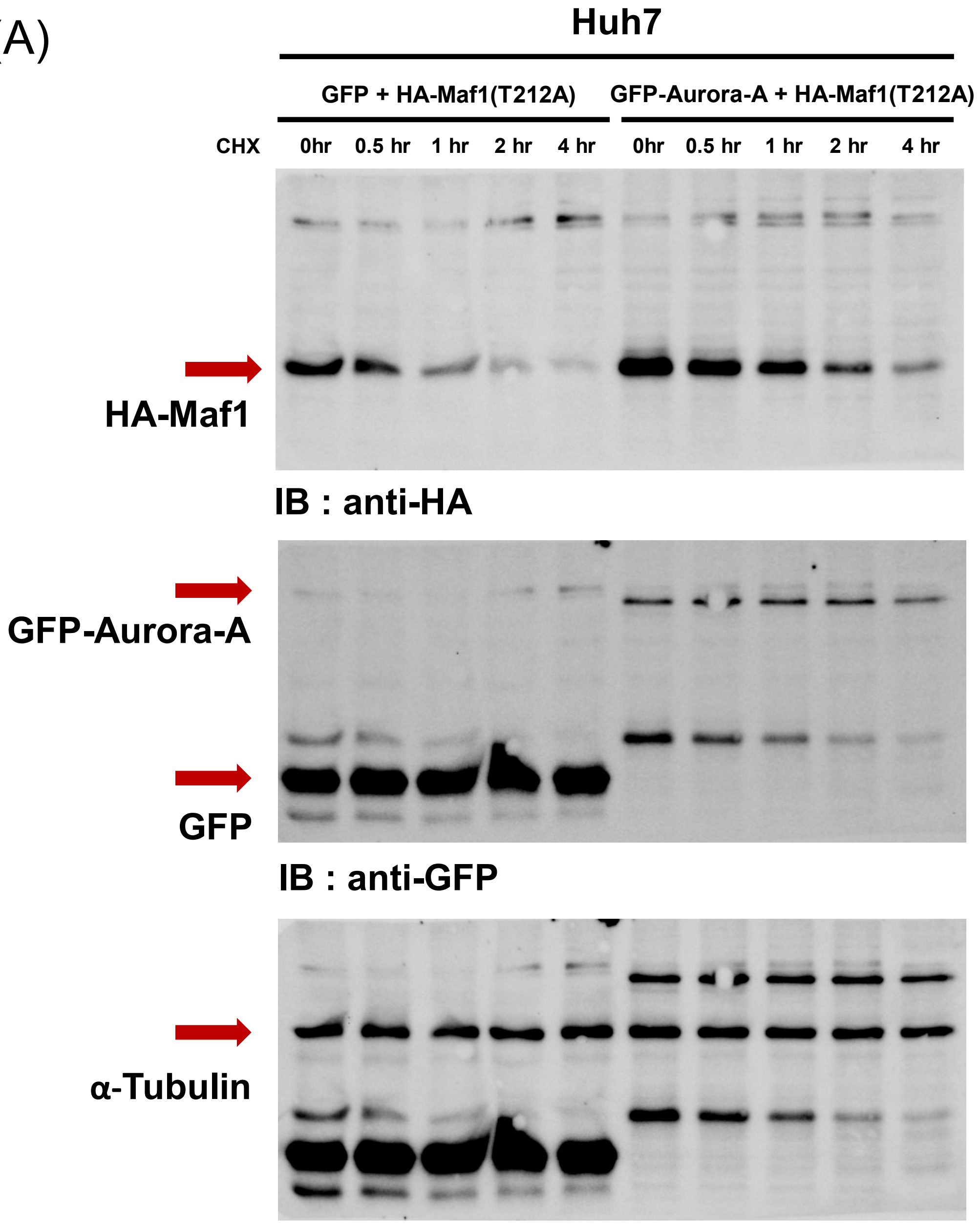

Supplement: Supplementary file 4 — Original Data [file 41420_2025_2885_MOESM4_ESM.pdf]
