# Supplementary figures and images for: Aurora-A-mediated cytosolic localization of Maf1 promotes cell proliferation via regulating mitochondrial function in HCC
Source: Cell Death Discov. 2025 Dec 3;11:561. doi: 10.1038/s41420-025-02885-z (PMC12717421; doi:10.1038/s41420-025-02885-z)

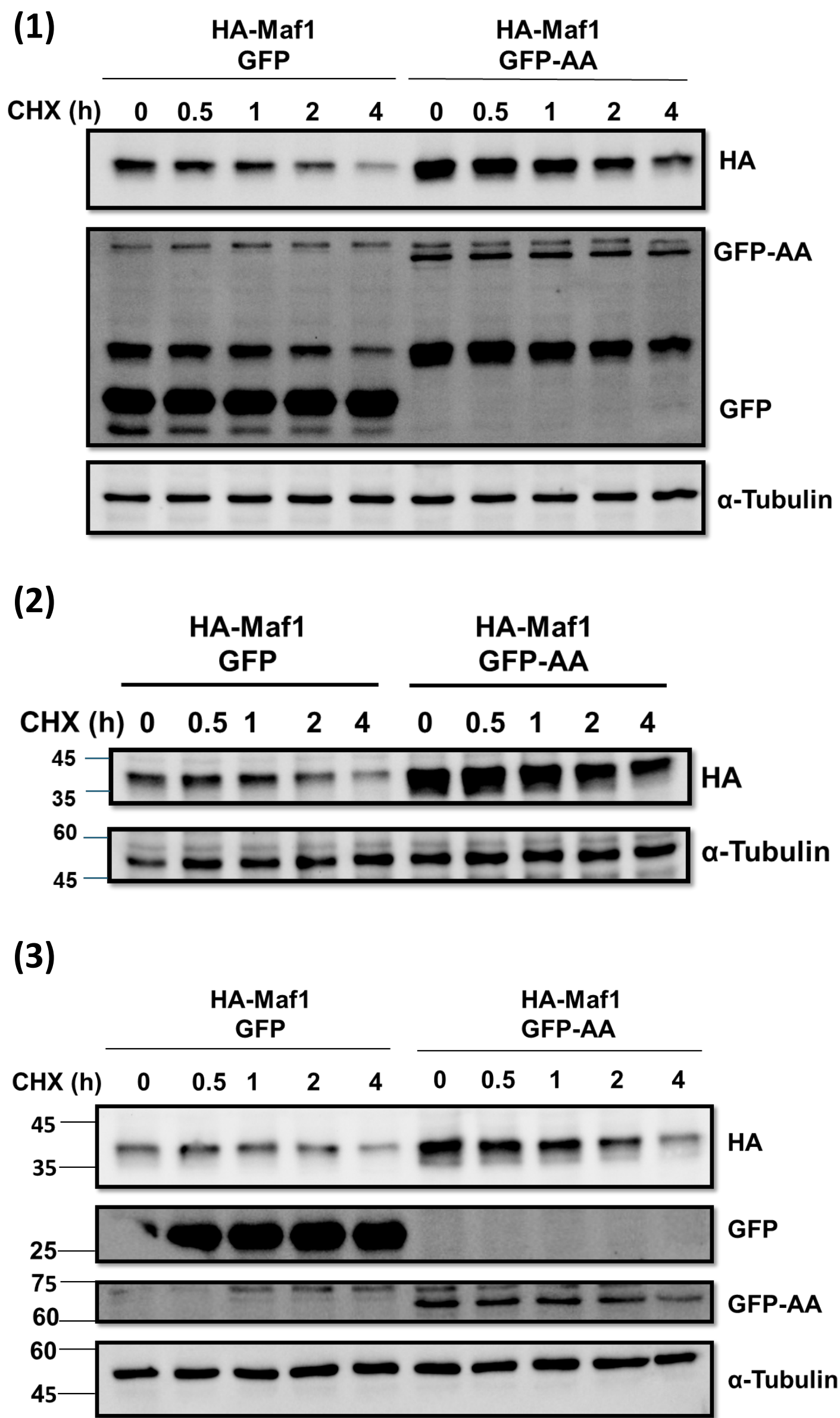

**(1)**

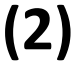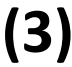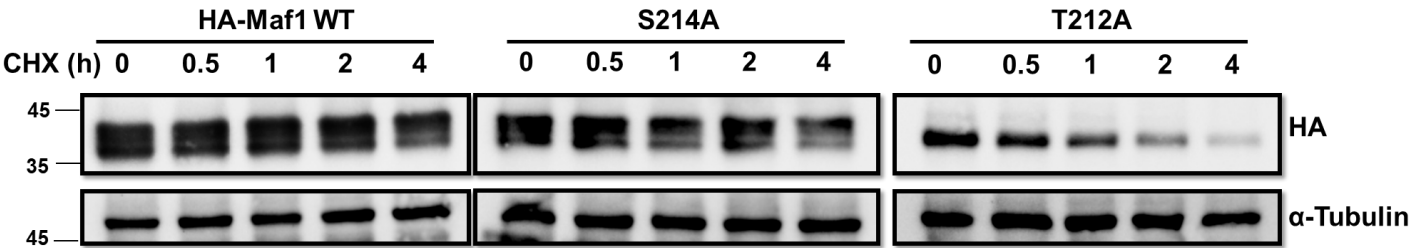

(1)

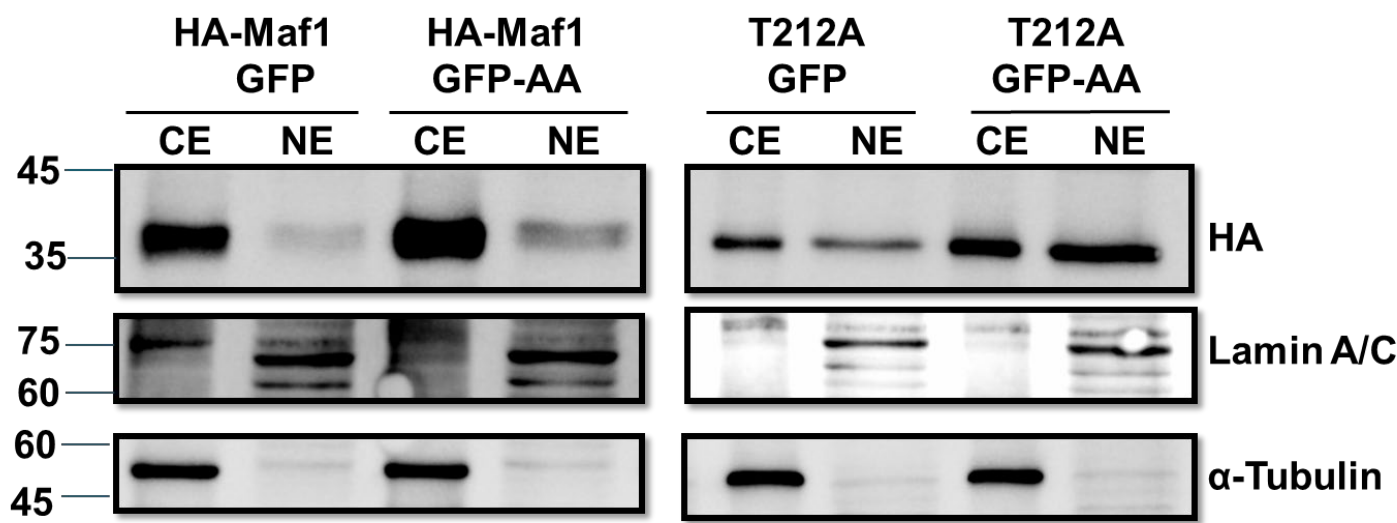

(2)

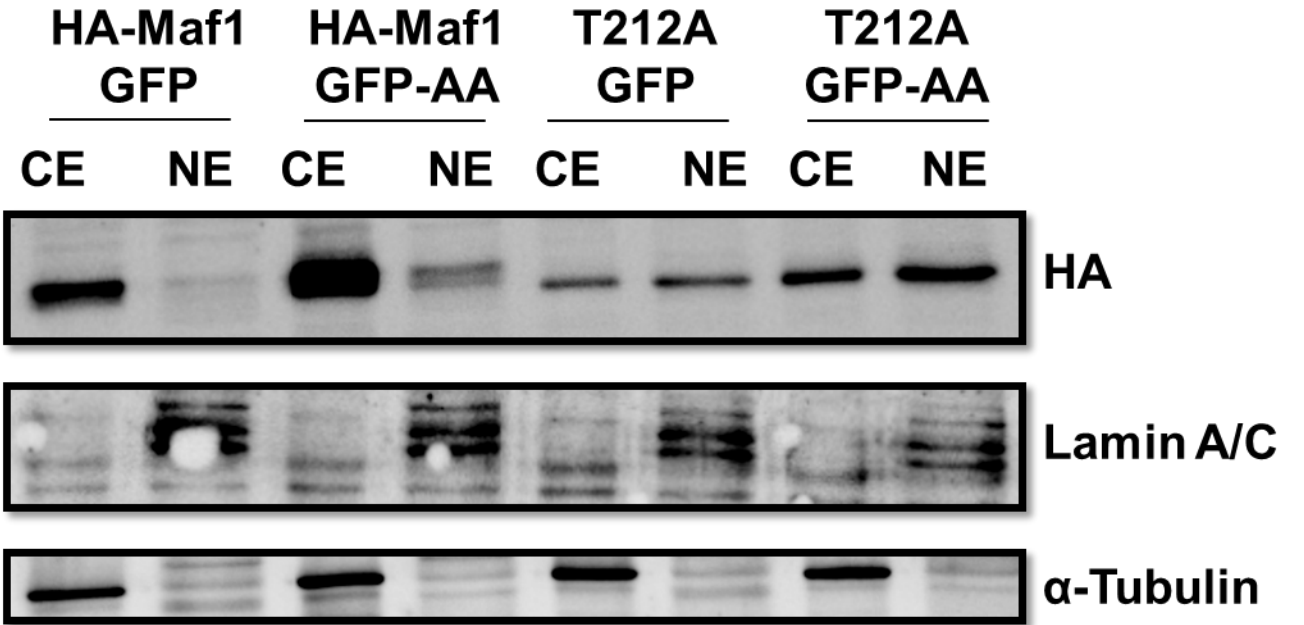

(3)

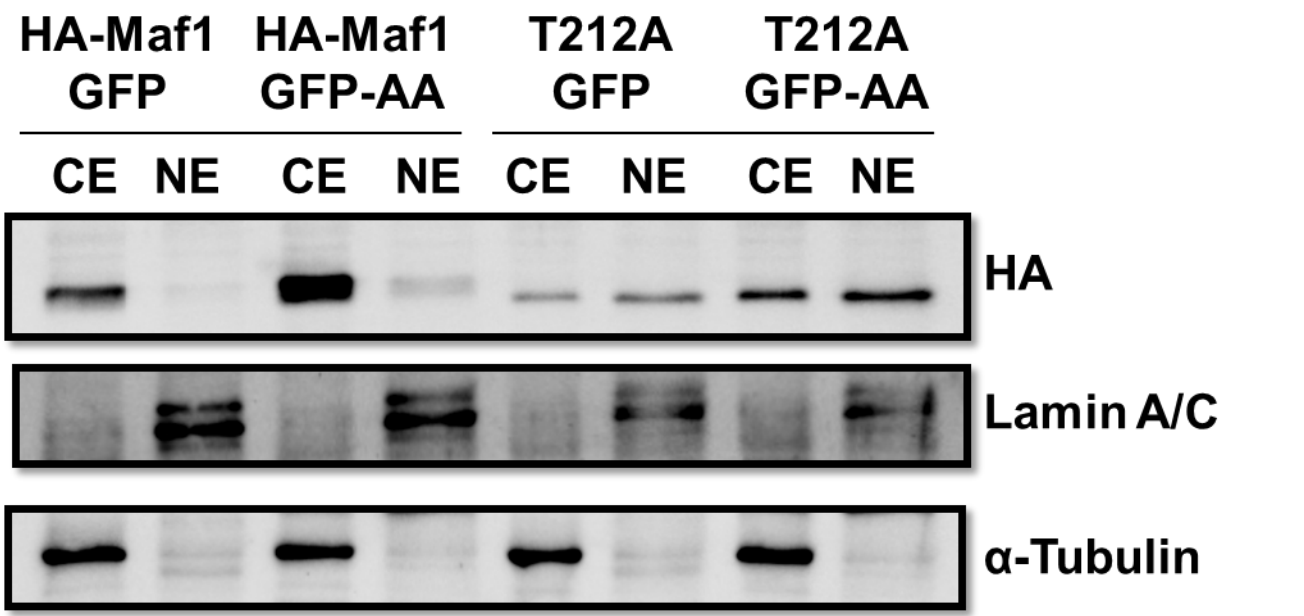

(1)

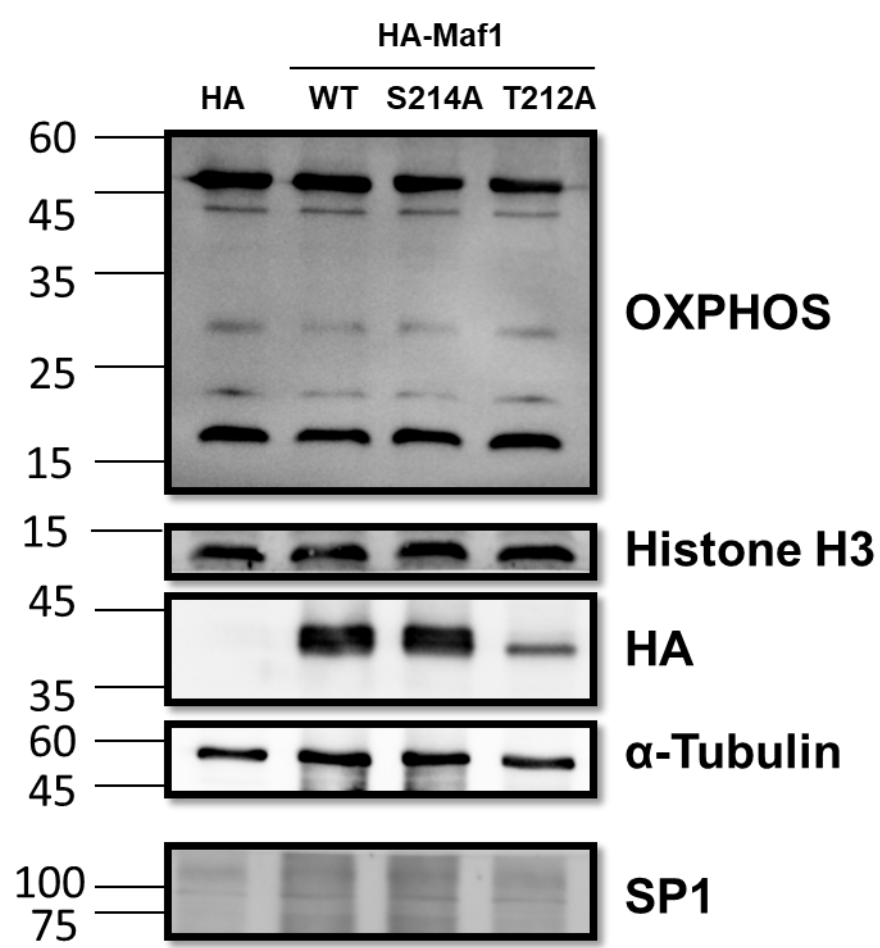

(2)

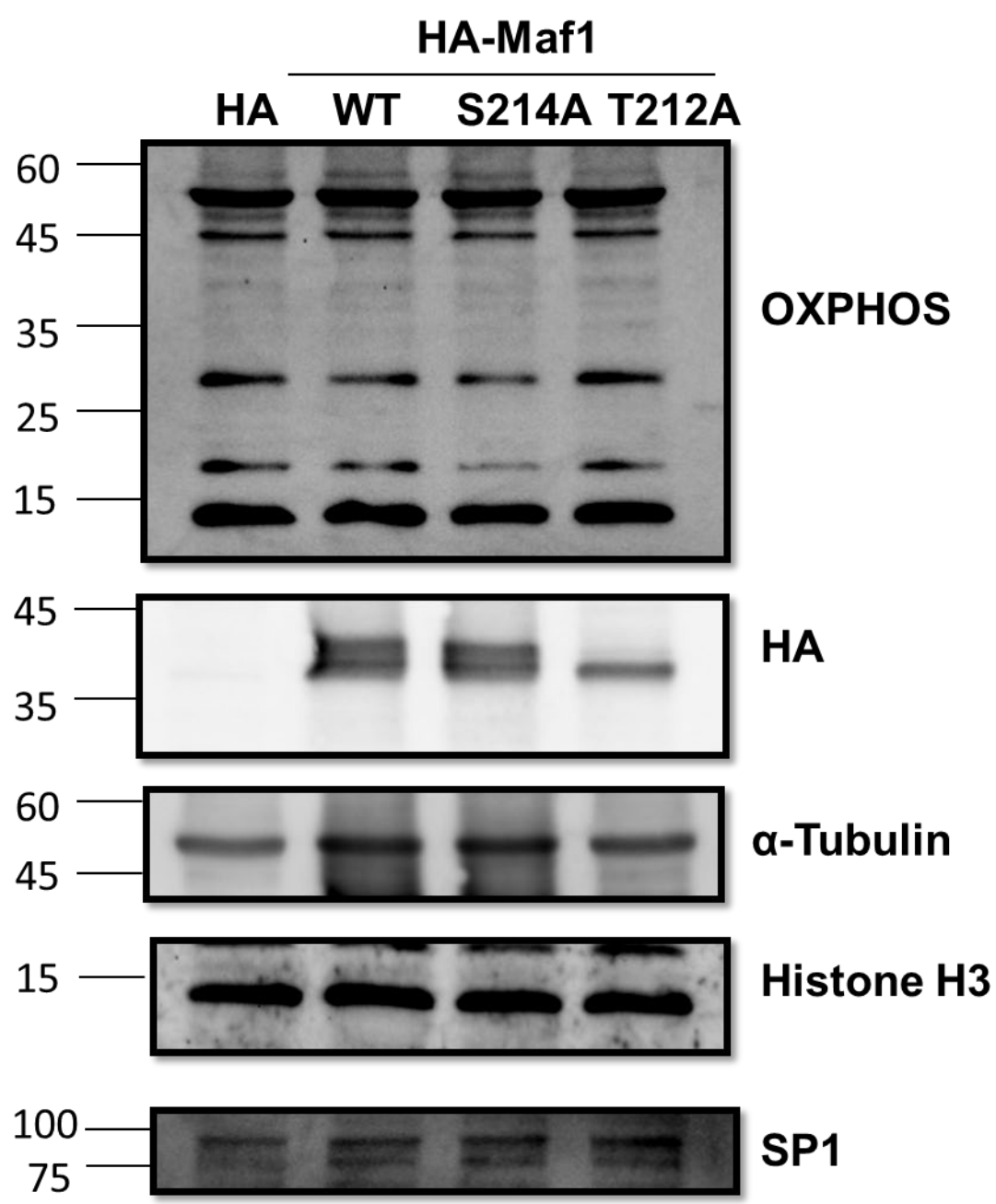

(3)

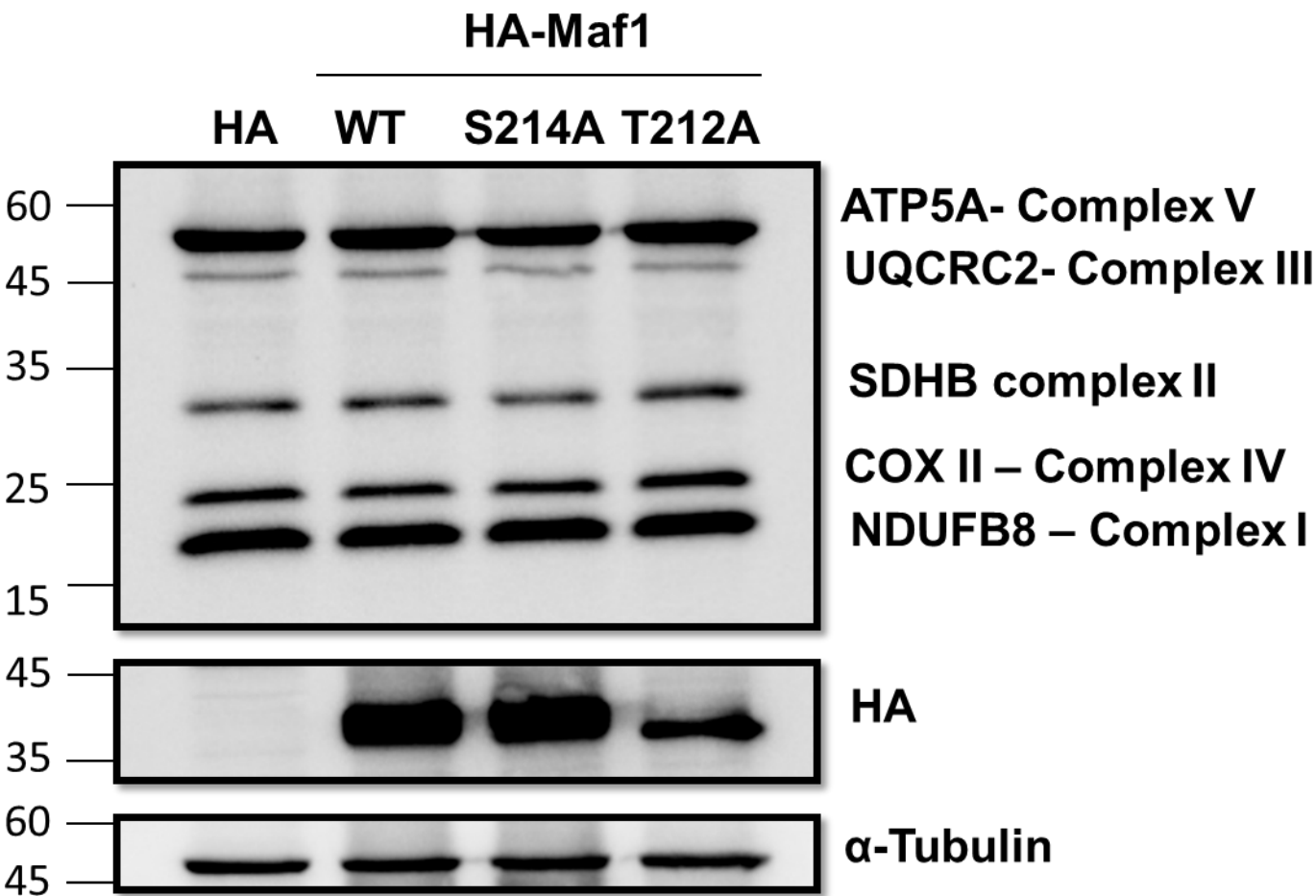

(1)

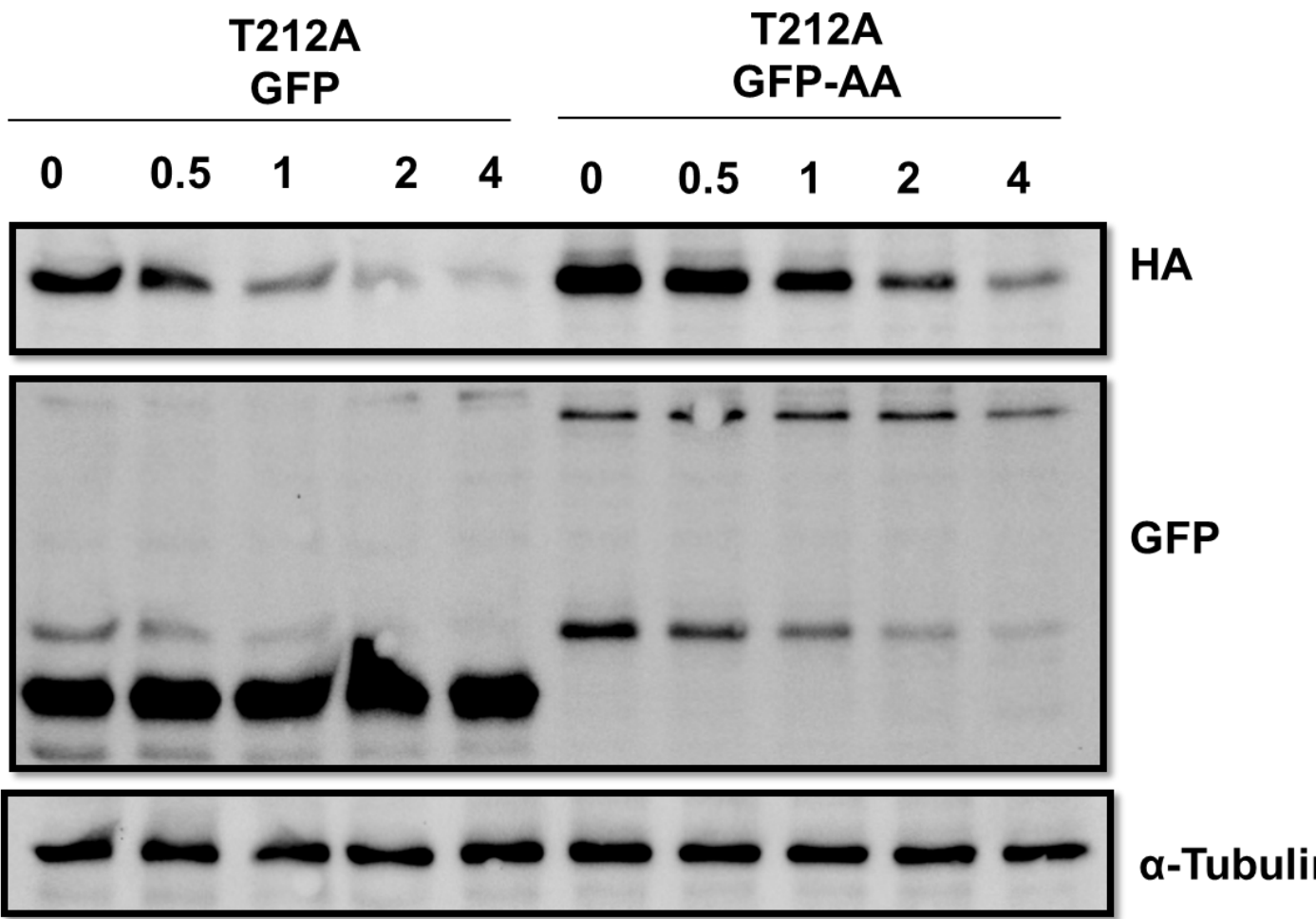

(2)

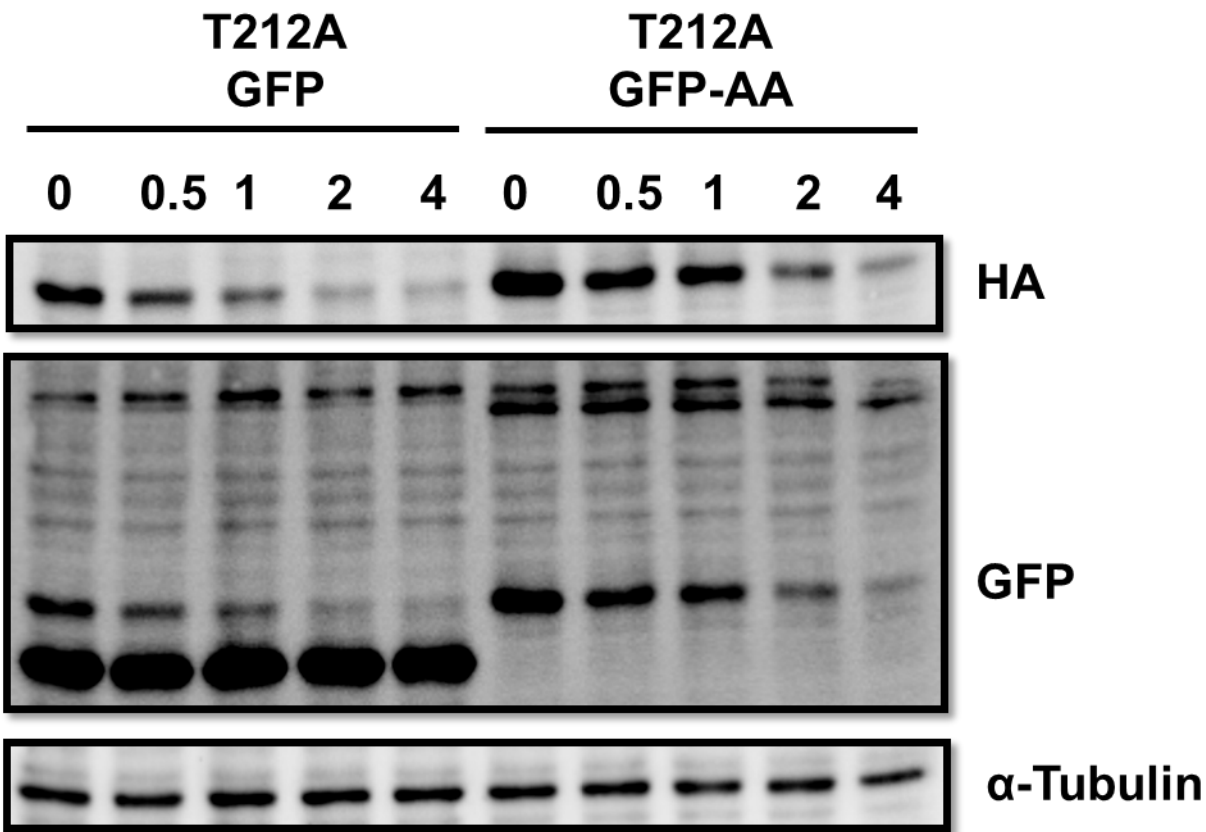

(3)

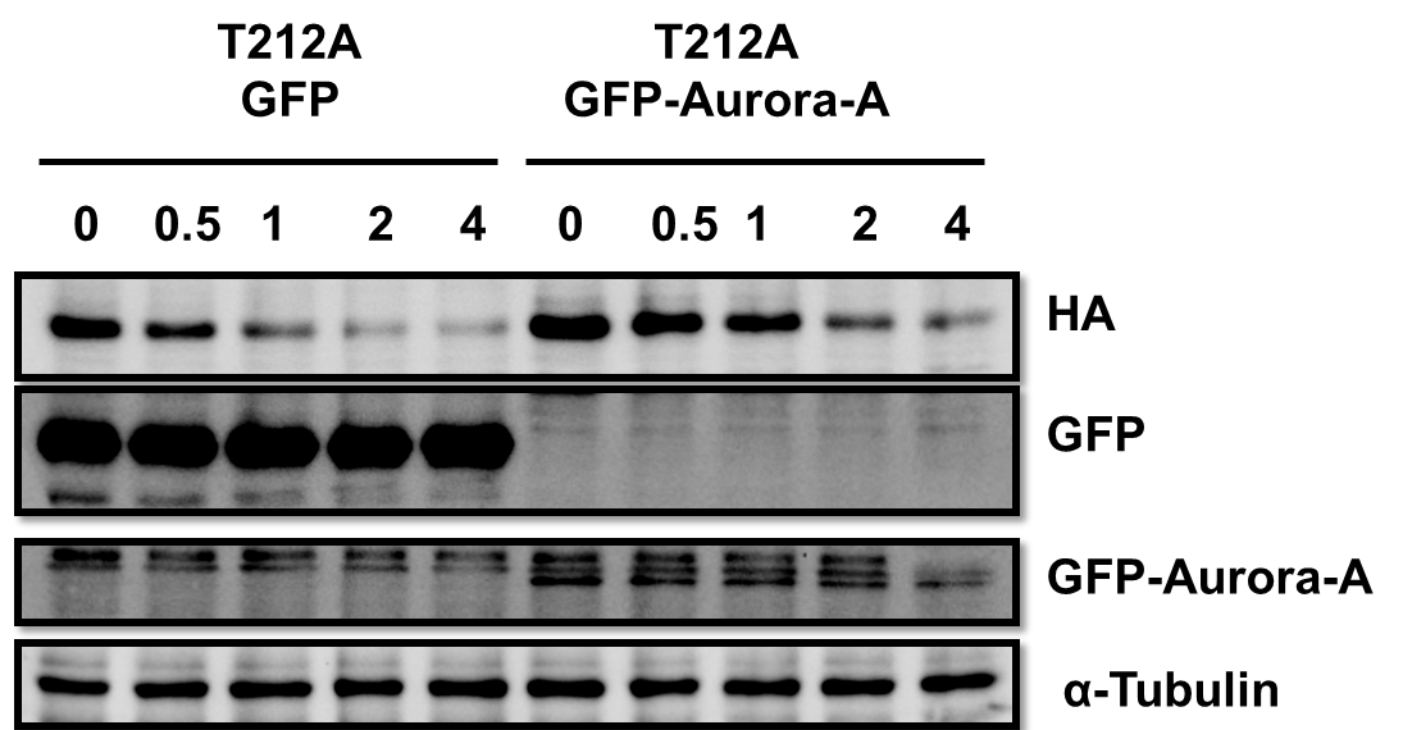

Supplement: Supplementary file 5 — Three independent experiments of western blot [file 41420_2025_2885_MOESM5_ESM.pdf]
